# Volatility of Tax Payments and Dividend Payouts*

**DOI:** 10.1111/1911-3846.12831

**Published:** 2022-12-31

**Authors:** Harald J. Amberger

**Affiliations:** ^1^ Vienna University of Economics and Business

**Keywords:** volatility of tax payments, cash ETR volatility, dividends, tax uncertainty, tax risk, real effects, irrégularité des paiements de l'impôt, instabilité des recettes á taux effectif, dividendes, incertitude fiscale, risque fiscal, effets réels

## Abstract

Dividends are a key mechanism for shareholders to discipline managers and mitigate agency conflicts. This study examines whether the volatility of tax payments is associated with dividend payouts. Consistent with the predictions, results suggest that firms with more volatile tax payments are less likely to pay dividends overall and their dividends are lower in magnitude when doing so. These effects are economically significant and incremental to a firm's operating risk. The link between volatile tax payments and the likelihood of dividend payouts is weaker for firms that distribute dividends to alleviate agency conflicts. Similarly, the link between volatile tax payments and the amount of dividend payouts is weaker for firms that hold more cash for tax reasons. Taken together, these findings add to our understanding of the economic consequences of volatile tax payments and the determinants of dividend payouts.

## RÉSUMÉ

Irrégularité dans les paiements de l'impôt et les versements de dividendes

Les dividendes représentent un mécanisme clé permettant aux actionnaires de discipliner les gestionnaires et d'atténuer les conflits entre mandant et mandataire. Cette étude examine si l'irrégularité des paiements de l'impôt est associée aux versements de dividendes. Conformément aux prédictions, les résultats suggèrent que les entreprises dont les paiements de l'impôt sont plus irréguliers sont généralement moins susceptibles de verser des dividendes et que leurs dividendes sont alors moins substantiels. Ces effets sont économiquement significatifs et augmentent le risque opérationnel de l'entreprise. Le lien entre l'irrégularité des paiements de l'impôt et la probabilité de verser des dividendes est plus faible pour les entreprises qui distribuent des dividendes afin d'atténuer les conflits entre mandant et mandataire. De même, le lien entre les paiements d'impôts irréguliers et le montant des versements de dividendes est plus faible pour les entreprises qui détiennent plus de liquidités pour des raisons fiscales. Dans l'ensemble, ces résultats améliorent notre compréhension des conséquences économiques de l'irrégularité des paiements de l'impôt et des facteurs déterminants des versements de dividendes.

## 1 Introduction

This study examines whether and the extent to which the volatility of tax payments is associated with dividend payouts. Prior research suggests that the volatility of tax payments can result in higher loan spreads (Saavedra 2019), which speaks to the consequences of volatile tax payments for debtholders. However, there is scant evidence of the link between the volatility of tax payments and payouts to equity holders. This gap is surprising because dividend payouts are an integral element of a firm's financial ecosystem (Farre‐Mensa et al. 2014).

Understanding the payout consequences of volatile tax payments is important because dividends are a key mechanism for shareholders to discipline managers and mitigate agency conflicts (Easterbrook 1984). Moreover, through its tax claim, the taxing government is a de facto silent shareholder in a firm (Desai et al. 2007). Thus, investigating the relation between volatile tax payments and dividend payouts not only informs our understanding of the drivers of dividend payouts but also sheds light on whether the volatility of the government's tax claim has implications for a firm's payouts to its *actual* shareholders.

Tax payments could be volatile due to managerial decisions. For example, a firm might claim risky or uncertain tax positions that are not upheld in a tax audit or in court. Alternatively, a firm could claim short‐lived tax benefits. The extent to which a firm relies on such tax positions shapes the volatility of tax payments over time (McGuire et al. 2013; Guenther et al. 2017; Drake et al. 2019). However, tax payments could also be volatile due to reasons beyond a firm's control, such as the required application of tax laws that are frequently subject to legislative change. Moreover, nontax factors, such as the riskiness of operations, could contribute to the volatility of tax payments because all income and expenses ultimately flow through a firm's tax accounts.

A firm's greater ability to control the volatility of tax payments is what distinguishes this risk from general cash flow risk (Saavedra 2019), which prior research has found to affect dividend payouts (Bradley et al. 1998; Chay and Suh 2009). Specifically, survey evidence suggests that managers can change tax positions rather quickly (Hoopes et al. 2012, Appendix A), while the general business strategy that shapes cash flow risk usually has a multiyear horizon (Hubbard 1998). Thus, for a given level of general cash flow risk, managers have some discretion over the extent to which they pursue risky tax strategies (Jacob et al. 2022). By examining the payout consequences of volatile tax payments, my study provides further insight into the factors that shape dividend policy.

In making a dividend payout decision, managers forecast cash requirements in order to avoid costly liquidity shortfalls and dividend reductions (Lintner 1956; Brav et al. 2005). Since volatile tax payments could increase the likelihood of experiencing a liquidity shortfall, the volatility of tax payments should be negatively associated with dividend payouts. Volatile tax payments could also lower the likelihood of maintaining consistent dividend levels over time, requiring costly future adjustments (Healy and Palepu 1988). Hence, for firms paying a dividend, I expect that more volatile tax payments are associated with a lower magnitude of dividend payouts. My predictions are not without tension, however. Prior research suggests that, in order to ensure consistent dividend payouts over time, managers cut back on investment (Brav et al. 2005) or raise capital externally (Farre‐Mensa et al. 2021). If managers are willing to incur the costs associated with these strategies, the volatility of tax payments will have little effect on dividend payouts. Thus, whether volatile tax payments are associated with dividend payouts is an empirical question.

I test my predictions on a sample of public US firms with financial statement data for fiscal years 1993–2014. I measure the volatility of these firms' tax payments using the coefficient of variation for annual cash effective tax rates (ETRs) over the prior five years (McGuire et al. 2013). Since the riskiness of operations and financial statement losses could contribute to volatile tax payments, I orthogonalize the measure by industry‐year with respect to proxies for operating risk and losses (Neuman et al. 2020). This procedure removes the impact of operating risk and losses from the measure, isolating volatility associated with a firm's tax strategy.^1^ The resulting measure aligns with empirical evidence that variation in cash ETRs is related to the future volatility of tax payments (Drake et al. 2019). Hence, managers could form expectations about the cash flow implications of a firm's tax strategy and incorporate this information in their dividend payout decisions. Because the measure is based on cash ETRs, it is also conceptually linked to dividends, which are paid using after‐tax cash flow.

My results suggest that volatile tax payments are associated with a lower likelihood of dividend payouts. In economic terms, a one standard deviation increase in the volatility of tax payments is associated with a 4.9 percentage point lower probability of a payout. This effect is incremental to that of operating cash flow volatility, consistent with tax payments being a significant cash flow component. In the subsample of dividend‐paying firms, I find a negative relation between volatile tax payments and the amount of dividends paid. A one standard deviation increase in the volatility of tax payments is associated with 3% lower dividend levels, translating into a lower payout of $6.8 million for the average dividend‐paying firm.

These results are robust to a battery of robustness tests. In particular, I find consistent results using alternative measures of the volatility of tax payments, which (i) exclude loss observations, (ii) are based on the standard deviation of annual cash ETRs (Guenther et al. 2017), (iii) exploit large tax payments (Saavedra 2019), and (iv) use annual GAAP instead of cash ETRs. Moreover, supplemental analyses mitigate concerns that operating risk might drive my findings. Finally, in addition to including firm fixed effects in tests of dividend levels, I further alleviate endogeneity concerns by estimating a changes specification. All tests provide consistent evidence that the volatility of tax payments is negatively associated with both the decision to pay dividends and the magnitude of those payouts. Furthermore, they suggest the effect of volatile tax payments on dividend payouts is incremental to operating risk.

I next conduct several cross‐sectional tests to explore how the relation between volatile tax payments and dividend payouts varies across firms. First, I exploit variation in the motives for paying a dividend. Dividends are a key mechanism to alleviate agency conflicts (Easterbrook 1984) and shareholders are willing to incur the costs of liquidity shortfalls in order to achieve the desired payout policy (Jensen 1986). Thus, I expect firms to be less sensitive to the volatility of tax payments when agency motives dominate a payout decision. My results support this prediction and suggest lower agency costs moderate the effect of volatile tax payments on the likelihood of dividend payouts. Second, I test whether the relation between volatile tax payments and dividend levels is weaker for firms with higher unrecognized tax benefits (UTBs). These firms tend to hold more cash for tax reasons (Hanlon et al. 2017), which should make it easier to maintain consistent dividend levels over time. I find support for this prediction, suggesting that sidelining more cash for tax reasons makes firms less sensitive to the volatility of tax payments in their dividend payout decisions. Finally, I provide empirical support for the theoretical arguments underlying my main predictions by showing that the relation between volatile tax payments and the probability of dividend payouts is stronger for firms with higher borrowing costs.^2^ With respect to dividend levels, I find a weaker relation for firms that incur lower costs for dividend reductions.

Additional tests suggest that besides having an incremental effect, the volatility of tax payments can also be incrementally informative about dividend payouts. Specifically, the negative relation between volatile tax payments and dividend payouts holds for different levels of cash flow risk while the volatility of operating cash flows provides no signal for firms with low cash flow risk. Furthermore, I find that the current volatility of tax payments can be informative about a firm's future dividend payouts and that firms with more volatile tax payments prefer more flexible distribution channels, such as share repurchases or special dividends.

This study makes several contributions to the literature. First, my results extend prior research on the economic consequences of volatile tax payments for investors. My finding that the volatility of tax payments is negatively associated with dividend payouts adds to the evidence that volatile tax payments are associated with higher loan spreads (Saavedra 2019). I also show that the relation between volatile tax payments and dividend payouts varies with the importance of agency motives for paying a dividend and the extent of cash held for tax reasons.

Second, my study adds to research on the determinants of dividend payouts. Although prior research finds that general cash flow risk is negatively related to dividend payouts (Chay and Suh 2009), I document a negative effect of volatile tax payments that is incremental to operating risk. I also quantify the effect of volatile tax payments on the probability and the amount of dividend payouts, and show that the volatility of tax payments can be incrementally informative. In providing these results, I respond to a call by Farre‐Mensa et al. (2014, 78) to use insights from fields outside of finance to advance our understanding of corporate payout policy.

## 2 Theoretical background and hypothesis development

### Determinants of dividend payouts

Dividends are a strategic tool in capital market economies, and prior research has identified several reasons that explain variation in payouts across firms. First, firms distribute dividends to alleviate agency conflicts between managers and shareholders (Officer 2011). Given excess cash, managers might divert funds (Jensen and Meckling 1976) or invest suboptimally (Jensen 1986). Dividends can constitute a disciplining device that forces managers to raise funds under external scrutiny (Easterbrook 1984).

Second, a firm could use dividends to convey information to capital market participants (Bhattacharya 1979; John and Williams 1985; Miller and Rock 1985). Because of information asymmetries between managers and shareholders, dividend payouts can reveal managers' private information and signal their earnings expectations to investors. Consistent with this argument, Grullon et al. (2002) find positive abnormal returns for dividend increases while investors tend to punish reductions in dividend payouts.

Third, the investor‐level tax on dividends relative to share repurchases could influence dividend policy (Poterba 2004). In support of this argument, Chetty and Saez (2005) document the largest increase in dividend payouts after the 2003 dividend tax cut (Jobs and Growth Tax Relief Reconciliation Act) for firms dominated by individual investors; that is, the investor group that was the main beneficiary of the reform.

Finally, the general business strategy can also shape a firm's dividend policy (Smith and Watts 1992). Hoberg et al. (2014), for instance, study product market fluidity and document lower dividend payouts for firms operating in more competitive industries. Along these lines, Bradley et al. (1998) and Chay and Suh (2009) investigate a risk‐based argument and find a negative relation between dividend payouts and general cash flow risk. Accordingly, DeAngelo et al. (2006) document a higher likelihood of dividend payouts for mature firms with high retained earnings but limited investment opportunities.

### Volatility of tax payments

Tax payments could be volatile due to managerial decisions. For example, a firm might claim risky or uncertain tax positions in one year that are reversed upon a tax audit in later years. If the IRS challenges a tax position that is subsequently not upheld in court, the firm could become liable for tax repayments, interest charges, and fines.^3^ Furthermore, a firm might rely on temporary tax benefits, such as short‐lived tax credits or tax incentives. If these tax benefits occur sporadically or are limited in time, they will reduce tax payments in single years but lead to higher tax payments in the future. In addition, the volatility of tax payments could be due to reasons beyond a firm's control. Tax laws are regularly subject to legislative change, which means that tax positions taken in one year could be infeasible in later years.

Aside from these tax reasons, nontax factors can also lead to volatile tax payments. Specifically, since all income and expenses ultimately flow through a firm's tax accounts, the general business strategy as well as the riskiness of operations could drive the volatility of tax payments. Hence, a firm's operating risk might influence the extent to which tax payments vary over time.

Several studies investigate the relation between volatile tax payments and overall firm risk. Guenther et al. (2017), for instance, show that the volatility of annual cash ETRs is positively related to variation in future stock returns. This result is consistent with the idea that volatile tax payments can reflect uncertainty about future cash flows. Similarly, Hutchens and Rego (2015) use a variety of proxies to measure overall firm risk and document a positive relation between the volatility of cash ETRs and (i) current stock return volatility, (ii) future stock return volatility, and (iii) analyst forecast dispersion.^4^ Collectively, these studies suggest that volatile tax payments are associated with greater overall firm risk.

Another stream of research examines economic consequences of volatile tax payments. Drake et al. (2019) find that equity investors positively value tax avoidance activities while the volatility of tax payments moderates this positive valuation. Jacob et al. (2022) focus on investment decisions and show that volatile tax payments are associated with a delay in major capital investments as well as lower investment levels overall. Most closely related to my study is Saavedra (2019), who studies the pricing of syndicated loans and finds a positive relation between tax volatility and loan spreads. Taken together, prior research shows that the volatility of tax payments can have economic consequences for the firm. However, these studies do not investigate the implications of volatile tax payments for payouts to equity holders.

An emerging body of research focuses on the risk embedded in a firm's tax strategy and uses a variety of measures to capture the constructs of tax risk and tax uncertainty.^5^ Neuman et al. (2020, 1789), for instance, define tax risk broadly as “the uncertainty about future tax outcomes generated by current actions or activities, or the failure to take actions or pursue activities.” Their practitioner‐based measure of tax risk is associated with a greater volatility of reported taxes and less persistent cash ETRs. Dyreng et al. (2019, 179) define tax uncertainty less broadly as “the potential loss of tax savings upon challenge.” Using UTBs as their measure of tax uncertainty, the authors find that firms with lower cash ETRs report greater additions to UTBs. Hanlon et al. (2017) build on this result and find that UTB balances are positively associated with tax‐based precautionary cash holdings.

### Hypothesis development

In making a dividend payout decision, managers forecast cash requirements to avoid liquidity shortfalls (Lintner 1956; Brav et al. 2005). Such shortfalls can be costly for a firm because they require raising capital externally in financial markets, which is more costly than internal financing through retained earnings (Myers and Majluf 1984). Anecdotal evidence indicates that managers are aware that more volatile tax payments could have a negative impact on their firm's after‐tax cash flows. According to an article in the *Financial Times*, 136 US firms, including LinkedIn Corp. and Yahoo! Inc., issued an alert in their 2015 financial statements that tax positions taken in the past could adversely affect future after‐tax earnings and cash flows (Houlder 2016). Since volatile tax payments could increase the likelihood of liquidity shortfalls, firms with more volatile tax payments should be less likely to distribute dividends.

Moreover, managers distribute dividends only if they are confident of maintaining consistent dividend levels over time. They tend to refrain from reducing payouts in the future (Lintner 1956; Brav et al. 2005). Since dividends allow investors to form expectations about a firm's earnings potential, reductions in dividend payouts might require investors to revise their expectations (Healy and Palepu 1988), leading to negative capital market reactions (Michaely et al. 1995; Grullon et al. 2002). In this regard, volatile tax payments could make it more difficult to achieve consistent dividend levels over time or increase the likelihood of future dividend reductions or omissions.

In sum, the volatility of tax payments could increase the likelihood of liquidity shortfalls and dividend reductions, potentially increasing costs in the form of external financing and capital market punishment. I therefore expect firms with more volatile tax payments to be less likely to pay a dividend. Moreover, when tax payments are volatile, managers might adjust dividend payouts to ensure consistent dividend levels over time, suggesting that the volatility of tax payments could also be associated with a lower amount of dividend payouts. Based on these arguments, I posit the following hypotheses:

Hypothesis 1 (H1) *The volatility of tax payments is negatively associated with the probability of dividend payouts*.

Hypothesis 2 (H2) *The volatility of tax payments is negatively associated with the amount of dividend payouts*.

Despite these predictions, I note that there is support for the null hypothesis. Prior research suggests that in order to ensure persistent dividend payouts over time, managers are willing to incur the costs associated with external financing (Farre‐Mensa et al. 2021) or forego valuable investment projects (Brav et al. 2005). If, due to volatile tax payments, a firm raises external capital or cuts back on investment instead of adjusting dividends, I will find no relation between the volatility of tax payments and dividend payouts.

## 3 Research design and data

### Variable measurement

To measure the volatility of tax payments (*CVTax*), I use the coefficient of variation for annual cash ETRs (McGuire et al. 2013).^6^ *CVTax* is calculated as the standard deviation of annual cash ETRs over the five‐year period *t* − 4 to *t*, scaled by the mean cash ETR measured over the same period.^7^ I orthogonalize *CVTax* with respect to the volatility of operating cash flows (*CVCashFlow*), pre‐tax income (*CVRoa*), and sales (*CVSales*), all measured over the five‐year period *t* − 4 to *t*, as well as an indicator for the presence of negative pre‐tax income in at least one of these years (*LossFirm*).^8^ This approach removes the impact of operating risk and losses from the measure and isolates the volatility of tax payments associated with a firm's tax strategy (Higgins et al. 2015).^9^ Consistent with Neuman et al. (2020), I orthogonalize *CVTax* per year and Fama and French 17 industry codes.^10^ The resulting measure captures firm‐level deviations in the volatility of tax payments from the average firm in an industry‐year, and is unaffected by industry characteristics or industry‐specific trends (Badertscher et al. 2019).^11^

*CVTax* is an appropriate measure in my setting for three reasons. First, Drake et al. (2019) show that the current volatility of cash ETRs is associated with variation in future tax payments. Hence, the volatility of tax payments allows managers to form expectations about the potential cash flow implications of variation associated with a firm's tax strategy and incorporate this information in a dividend payout decision. Second, a measure based on cash ETRs is conceptually linked to dividend payouts, as compared to income‐based measures such as GAAP ETRs. Tax payments map into after‐tax cash flows, from which dividends are paid. Third, a long‐term measure alleviates concerns that tax positions taken in year *t* could jointly affect the volatility of tax payments and dividend payouts (Klassen and Laplante 2012).

### Research design

To test H1 and H2, I estimate the following firm‐level regression model:

(1) $$\begin{array}{ll} {\text{DivPay}}_{\mathrm{it}}\;\left({\text{DivAmount}}_{\mathrm{it}}\right)=\alpha & +\beta_{1}{\text{CVTax}}_{\mathrm{it}-4,t}+\beta_{2}{\text{TaxAvoidance}}_{\mathrm{it}-4,t}+\beta_{3}{\text{CashFlow}}_{\mathrm{it}}\\ & +\beta_{4}{\text{CVCashFlow}}_{\mathrm{it}-4,t}+\beta_{5}{\text{CVRoa}}_{\mathrm{it}-4,t}+\beta_{6}{\text{CVSales}}_{\mathrm{it}-4,t}+\beta_{7}{\text{Cash}}_{\mathrm{it}}\\ & +\beta_{8}{\mathrm{MTB}}_{\mathrm{it}}+\beta_{9}\mathrm{Sa}l{\text{esGrowth}}_{\mathrm{it}-2,t}+\beta_{10}{\text{AssetGrowth}}_{\mathrm{it}}+\beta_{11}{\text{RETE}}_{\mathrm{it}}\\ & +\beta_{12}{\mathrm{Age}}_{\mathrm{it}}+\beta_{13}{\text{Size}}_{\mathrm{it}}+\beta_{14}{\text{Leverage}}_{\mathrm{it}}+\beta_{15}{\mathrm{PCM}}_{\mathrm{it}}+\beta_{16}{\text{Loss}}_{\mathrm{it}}\\ & +\beta_{17}{\mathrm{NOL}}_{\mathrm{it}}+\beta_{18}R\&D_{\mathrm{it}}+\beta_{19}{\mathrm{SGA}}_{\mathrm{it}}+\beta_{20}{\text{Advertising}}_{\mathrm{it}}\\ & +\beta_{21}\text{CapInt}e{\text{nsity}}_{\mathrm{it}}+\text{FixedEffects}+\varepsilon_{\mathrm{it}}. \end{array}$$

I use a logit regression to examine the probability of dividend payouts (H1) and a linear regression to examine dividend levels (H2). Separate tests for the probability and the amount of dividend payouts are appropriate because the decision to pay a dividend is likely to precede decisions on their amount (Humphreys 2013).^12^ ^,^ ^13^ The dependent variable in the logit regression, *DivPay*, is an indicator variable equal to one if a firm declares dividends on common stock in year *t*, and zero otherwise (Hail et al. 2014). In the linear regression, I use the continuous variable *DivAmount*, which is the logarithm of dividends declared on common stock in year *t* divided by total assets (Hoberg et al. 2014).^14^

$\beta_{1}$ is the coefficient of interest. I expect $\beta_{1}<0$ in both tests, consistent with volatile tax payments being associated with (i) a lower probability and (ii) a smaller amount of dividend payouts. To control for tax avoidance, I calculate long‐run cash ETRs in the spirit of Dyreng et al. (2008). I multiply *CashETR* by negative one in all regressions so that larger values indicate more tax avoidance (*TaxAvoidance*).

I control for several determinants of dividend payouts. *CashFlow* is operating cash flows (adjusted for cash taxes paid), scaled by lagged total assets and controls for internal funds. Fama and French (2001) find a positive association between dividend payouts and a firm's internal funds. *CVCashFlow*, *CVRoa*, and *CVSales* control for operating risk, which should be negatively associated with dividend payouts.^15^ ^,^ ^16^ I also control for investment opportunities by including *Cash* as cash holdings divided by total assets, *MTB* as the market‐to‐book ratio, *SalesGrowth* as three‐year sales growth, and *AssetGrowth* as growth in total assets. Firms with greater investment opportunities require more cash, which should reduce the distribution potential (Fama and French 2001; Jacob and Jacob 2013). To control for the financial life cycle of a firm (DeAngelo et al. 2006), I include *RETE* as retained earnings divided by shareholder equity, *Age* as the logarithm of firm age, and *Size* as the logarithm of total assets. *Leverage* is long‐term debt divided by total assets. Since creditors mitigate agency conflicts between managers and shareholders, leverage should reduce the relevance of dividend payouts (Jensen 1986). *PCM* is the price–cost margin and controls for a firm's product market power (Kubick et al. 2015), which prior research has found to be positively associated with dividend payouts (Hoberg et al. 2014). *Loss* is an indicator variable equal to one if a firm reports negative net income, and zero otherwise, capturing the impact of financial statement losses (DeAngelo et al. 1992).

I also include *NOL* as the amount of net operating loss carryforward, *R&D* as R&D expense, *SGA* as SG&A expense, *Advertising* as advertising expense, and *CapIntensity* as capital intensity (all scaled by lagged total assets). These items could be associated with the volatility of cash ETRs and dividend payouts.

I would ideally include year and firm fixed effects in both regression models to capture year shocks and to control for time‐invariant firm characteristics that might drive the relationship between the volatility of tax payments and dividend payouts.^17^ However, due to the incidental parameters problem, estimates from a logit regression with firm fixed effects could be subject to an upward bias (Greene 2004; Allison 2009). I, therefore, include year and firm fixed effects only in tests of dividend levels but use year and industry fixed effects when investigating the probability of dividend payouts.^18^ ^,^ ^19^

To account for serial correlation in the data (Petersen 2009), I cluster standard errors by firm in tests of the probability of dividend payouts and by industry in tests of dividend levels.^20^ Continuous variables are winsorized at the 1st and 99th percentiles to mitigate the influence of outliers. I standardize all independent variables to have a mean of zero and a standard deviation of one prior to estimating regressions. This approach facilitates the comparison of effect sizes across specifications because each estimate reflects the impact of a one standard deviation change in the independent variable on dividend payouts (Wooldridge 2020).^21^

### Sample selection

I obtain firm‐year observations for fiscal years 1993–2014 from Compustat Industrial.^22^ The sample effectively covers fiscal years 1998–2014 because several variables require five years of data. I require firms to be incorporated in the United States.^23^ Moreover, I drop financial firms (SIC codes 6000–6999) and utilities (SIC codes 4900–4949) because these industries exhibit distinct accounting rules (McGuire et al. 2013) and payout patterns (Fama and French 2001). I also drop firms with names ending in “LP” or “TRUST” to remove flow‐through entities not subject to firm‐level taxes (Dyreng et al. 2008). Following prior research (Chay and Suh 2009; Hoberg et al. 2014), I exclude observations with dividends greater than sales, negative dividends, negative sales, and book equity below $250,000 or total assets below $500,000. Finally, I drop observations missing data needed to calculate the regression variables.

These restrictions yield a full sample of dividend‐paying and non‐dividend‐paying firms, including 33,023 firm‐year observations (4,569 unique firms). To obtain a subsample of dividend‐paying firms, I exclude observations with zero dividend payouts, resulting in 13,694 firm‐year observations (1,843 unique firms). Table 1 summarizes the sample selection.

**TABLE 1:** Sample selection

Data restrictions	Firm‐years	Firms
Observations in Compustat Industrial for fiscal years 1993–2014	251,072	28,169
*Less*: Observations of firms incorporated outside the United States	(54,052)	(6,442)
*Less*: Observations of financial firms (SIC codes 6000–6999) and utilities (SIC codes 4900–4949)	(63,697)	(7,238)
*Less*: Observations of firms with names ending in “LP” or “TRUST”	(1,427)	(161)
*Less*: Observations with dividends greater than sales	(1,857)	(522)
*Less*: Observations with negative dividends and negative sales	(5)	—
*Less*: Observations with book equity below $250,000 or total assets below $500,000	(21,539)	(681)
*Less*: Observations with insufficient data to compute *CVTax*	(59,412)	(6,360)
*Less*: Observations with insufficient data to compute control variables	(16,060)	(2,196)
Full sample: Dividend‐paying and non‐dividend‐paying firms	33,023	4,569
*Less*: Observations with zero dividend payouts	(19,329)	(2,726)
Subsample: Dividend‐paying firms	13,694	1,843

*Notes*: This table outlines the sample selection. The full sample (subsample) includes dividend‐paying and non‐dividend‐paying firms (dividend‐paying firms). Variables are defined in the Appendix.

### Descriptive statistics

Table 2 presents the descriptive statistics. Panel A shows information for the full sample. Panels B and C present information separately for dividend‐paying and non‐dividend‐paying firms; 41.5% of the firm‐years report dividend payouts (panel A), which amount to 2.7% of total assets for the average dividend‐paying firm (panel B). The mean of *CVTax* prior to orthogonalization is 0.71, which is slightly smaller than in McGuire et al. (2013). In line with H1, *CVTax* is significantly lower for dividend‐paying firms (*p* < 0.01).^24^ The average firm has a 28.4% *CashETR*, which is comparable to prior research (Guenther et al. 2017).

**TABLE 2:** Descriptive statistics and correlations

**Panel A:** Descriptive statistics for the full sample						
	*N*	Mean	SD	25%	Median	75%
*DivPay*	33,023	0.415	0.493	0.000	0.000	1.000
*CVTax*	33,023	0.713	0.493	0.315	0.619	0.994
*CashETR*	33,023	0.284	0.197	0.353	0.268	0.164
*CashFlow*	33,023	0.140	0.111	0.070	0.127	0.195
*CVCashFlow*	33,023	0.588	1.141	0.215	0.392	0.729
*CVRoa*	33,023	0.778	4.297	0.231	0.520	1.192
*CVSales*	33,023	0.198	0.160	0.090	0.151	0.253
*Cash*	33,023	0.149	0.163	0.026	0.086	0.220
*MTB*	33,023	2.683	2.637	1.188	1.915	3.149
*SalesGrowth*	33,023	0.230	0.433	−0.010	0.149	0.363
*AssetGrowth*	33,023	0.110	0.263	−0.018	0.060	0.165
*RETE*	33,023	0.350	1.081	0.146	0.527	0.828
*Age*	33,023	2.850	0.677	2.303	2.890	3.466
*Size*	33,023	6.301	1.977	4.949	6.295	7.614
*Leverage*	33,023	0.165	0.164	0.003	0.133	0.272
*PCM*	33,023	−0.017	0.120	−0.088	−0.026	0.037
*Loss*	33,023	0.176	0.381	0.000	0.000	0.000
*NOL*	33,023	0.062	0.165	0.000	0.000	0.037
*R&D*	33,023	0.028	0.050	0.000	0.000	0.034
*SGA*	33,023	0.288	0.230	0.117	0.238	0.401
*Advertising*	33,023	0.013	0.032	0.000	0.000	0.009
*CapIntensity*	33,023	0.559	0.407	0.242	0.458	0.784

**Panel B:** Descriptive statistics for dividend‐paying firms						
	*N*	Mean	SD	25%	Median	75%
*DivAmount*	13,694	0.027	0.035	0.009	0.016	0.030
*CVTax*	13,694	0.541	0.417	0.222	0.423	0.751
*CashETR*	13,694	0.283	0.146	0.348	0.283	0.205
*CashFlow*	13,694	0.154	0.099	0.089	0.140	0.204
*CVCashFlow*	13,694	0.410	0.414	0.171	0.285	0.486
*CVRoa*	13,694	0.632	1.877	0.196	0.385	0.755
*CVSales*	13,694	0.151	0.112	0.072	0.119	0.193
*Cash*	13,694	0.121	0.136	0.023	0.071	0.169
*MTB*	13,694	2.996	2.892	1.398	2.145	3.457
*SalesGrowth*	13,694	0.164	0.316	−0.010	0.117	0.269
*AssetGrowth*	13,694	0.081	0.195	−0.015	0.049	0.128
*RETE*	13,694	0.751	0.696	0.488	0.773	0.978
*Age*	13,694	3.175	0.612	2.773	3.367	3.664
*Size*	13,694	7.124	1.985	5.800	7.137	8.450
*Leverage*	13,694	0.177	0.149	0.028	0.164	0.275
*PCM*	13,694	0.002	0.110	−0.066	−0.010	0.048
*Loss*	13,694	0.098	0.298	0.000	0.000	0.000
*NOL*	13,694	0.030	0.076	0.000	0.000	0.019
*R&D*	13,694	0.017	0.032	0.000	0.000	0.021
*SGA*	13,694	0.252	0.210	0.095	0.202	0.353
*Advertising*	13,694	0.013	0.031	0.000	0.000	0.011
*CapIntensity*	13,694	0.640	0.405	0.325	0.566	0.891

**Panel C:** Descriptive statistics for non‐dividend‐paying firms						
	*N*	Mean	SD	25%	Median	75%
*DivAmount*	19,329	0.000	0.000	0.000	0.000	0.000
*CVTax*	19,329	0.836***	0.505	0.448	0.765	1.139
*CashETR*	19,329	0.284	0.226	0.360	0.252	0.129
*CashFlow*	19,329	0.130***	0.118	0.057	0.115	0.188
*CVCashFlow*	19,329	0.707***	1.732	0.274	0.504	0.918
*CVRoa*	19,329	0.886***	6.045	0.278	0.684	1.589
*CVSales*	19,329	0.237***	0.208	0.109	0.180	0.297
*Cash*	19,329	0.170***	0.177	0.028	0.102	0.262
*MTB*	19,329	2.467***	2.463	1.054	1.750	2.922
*SalesGrowth*	19,329	0.278***	0.505	−0.010	0.184	0.437
*AssetGrowth*	19,329	0.132***	0.303	−0.021	0.072	0.196
*RETE*	19,329	0.034***	1.476	−0.034	0.334	0.634
*Age*	19,329	2.620***	0.625	2.079	2.639	3.091
*Size*	19,329	5.720***	1.762	4.505	5.753	6.930
*Leverage*	19,329	0.158***	0.173	0.000	0.102	0.269
*PCM*	19,329	−0.030***	0.127	−0.103	−0.039	0.027
*Loss*	19,329	0.231***	0.421	0.000	0.000	0.000
*NOL*	19,329	0.086***	0.221	0.000	0.000	0.060
*R&D*	19,329	0.036***	0.059	0.000	0.000	0.056
*SGA*	19,329	0.314***	0.240	0.137	0.265	0.432
*Advertising*	19,329	0.014	0.035	0.000	0.000	0.008
*CapIntensity*	19,329	0.502***	0.400	0.199	0.387	0.702

**Panel D:** Correlation table for the full sample													
Variables	(1)	(2)	(3)	(4)	(5)	(6)	(7)	(8)	(9)	(10)	(11)	(12)	(13)
(1) *DivPay*	1.00												
(2) *CVTax*	**−0.29**	1.00											
(3) *CashETR*	0.00	**−0.29**	1.00										
(4) *CashFlow*	**0.11**	**−0.23**	**−0.11**	1.00									
(5) *CVCashFlow*	**−0.13**	**0.18**	**0.07**	**−0.13**	1.00								
(6) *CVRoa*	**−0.03**	**0.07**	0.01	**−0.03**	**0.04**	1.00							
(7) *CVSales*	**−0.24**	**0.28**	**0.05**	**−0.12**	**0.16**	**0.06**	1.00						
(8) *Cash*	**−0.15**	**0.08**	**−**0.01	**0.20**	**0.06**	0.01	**0.21**	1.00					
(9) *MTB*	**0.09**	**−0.12**	**−0.10**	**0.38**	**−0.08**	**−0.03**	**−0.06**	**0.14**	1.00				
(10) *SalesGrowth*	**−0.12**	**0.04**	**−0.14**	**0.24**	−0.01	0.01	**0.15**	−0.01	**0.14**	1.00			
(11) *AssetGrowth*	**−0.09**	0.00	**−0.13**	**0.27**	**−0.02**	0.01	**0.05**	**0.05**	**0.15**	**0.48**	1.00		
(12) *RETE*	**0.29**	**−0.33**	0.01	**0.19**	**−0.11**	0.01	**−0.21**	**−0.06**	−0.01	0.00	**0.03**	1.00	
(13) *Age*	**0.40**	**−0.14**	**−0.03**	**−0.02**	**−0.06**	**−0.01**	**−0.24**	**−0.12**	0.01	**−0.16**	**−0.10**	**0.19**	1.00
(14) *Size*	**0.34**	**−0.25**	**−0.14**	**0.06**	**−0.18**	**−0.05**	**−0.16**	**−0.20**	**0.19**	**0.03**	**0.05**	**0.22**	**0.27**
(15) *Leverage*	**0.06**	**0.05**	**−0.07**	**−0.16**	**−0.07**	−0.01	**−0.02**	**−0.43**	**0.07**	**0.05**	**0.06**	**−0.09**	0.01
(16) *PCM*	**0.13**	**−0.10**	**−0.23**	**0.48**	**−0.16**	**−0.03**	**−0.03**	−0.01	**0.23**	**0.23**	**0.19**	**0.16**	**0.03**
(17) *Loss*	**−0.17**	**0.22**	**0.21**	**−0.36**	**0.11**	**0.04**	**0.19**	−0.01	**−0.16**	**−0.20**	**−0.23**	**−0.24**	**−0.10**
(18) *NOL*	**−0.14**	**0.23**	**−0.07**	**−0.10**	**0.10**	0.01	**0.12**	**0.09**	0.01	−0.01	**0.04**	**−0.38**	**−0.03**
(19) *R&D*	**−0.18**	**0.14**	**−0.07**	**0.05**	**0.06**	**0.01**	**0.11**	**0.43**	**0.17**	**0.06**	**0.13**	**−0.12**	**−0.08**
(20) *SGA*	**−0.13**	−0.01	**0.10**	**0.14**	**0.07**	0.00	**−0.09**	**0.15**	**0.14**	**0.04**	**0.13**	**−0.05**	**−0.11**
(21) *Advertising*	0.00	**−0.07**	**0.04**	**0.12**	−0.01	−0.01	**−0.08**	**0.02**	**0.13**	0.00	**0.03**	**0.06**	**−0.03**
(22) *CapIntensity*	**0.17**	**0.05**	**0.08**	**0.21**	**−0.09**	0.01	**−0.14**	**−0.30**	**−0.02**	**0.03**	**0.13**	**0.09**	**0.11**

**Panel D:** Correlation table for the full sample									
Variables	(14)	(15)	(16)	(17)	(18)	(19)	(20)	(21)	(22)
(14) *Size*	1.00								
(15) *Leverage*	**0.32**	1.00							
(16) *PCM*	**0.28**	**0.16**	1.00						
(17) *Loss*	**−0.13**	**0.06**	**−0.33**	1.00					
(18) *NOL*	**−0.13**	**−0.02**	**−0.12**	**0.12**	1.00				
(19) *R&D*	**−0.11**	**−0.26**	**−0.05**	**0.06**	**0.19**	1.00			
(20) *SGA*	**−0.32**	**−0.28**	**−0.23**	0.00	**0.07**	**0.25**	1.00		
(21) *Advertising*	0.00	**−0.05**	0.00	**−0.03**	**−0.03**	**−0.07**	**0.41**	1.00	
(22) *CapIntensity*	**0.07**	**0.23**	**0.34**	**−0.08**	**−0.05**	**−0.23**	**−0.19**	−0.01	1.00

*Notes*: This table presents descriptive statistics (panels A–C) and pairwise correlation coefficients (panel D). Panel A presents descriptive statistics for the full sample of dividend‐paying and non‐dividend‐paying firms, panel B for dividend‐paying firms, and panel C for non‐dividend‐paying firms. *DivAmount* in panel B is dividends declared on common stock divided by total assets in year *t*. The variables in panels A–C are not standardized. I conduct two‐sample *t*‐tests to compare means between subsamples. *** represents a significance level of 0.01 (two‐tailed). Panel D presents Pearson correlation coefficients for the full sample. Bold coefficients represent significance levels of 0.01. All continuous variables are winsorized at the 1st and 99th percentile. Variables are defined in the Appendix.

Compared to non‐dividend‐paying firms, dividend‐paying firms exhibit lower operating risk (*CVCashFlow*, *CVRoa*, and *CVSales*), smaller cash holdings (*Cash*), lower sales growth (*SalesGrowth*), lower asset growth (*AssetGrowth*), a smaller net operating loss carryforward (*NOL*), lower R&D expense (*R&D*), lower SG&A expense (*SGA*), and are less likely to report a loss (*Loss*, all *p* < 0.01). Moreover, dividend‐paying firms have larger cash flows (*CashFlow*), higher market‐to‐book ratios (*MTB*), more retained earnings (*RETE*), more long‐term debt (*Leverage*), higher product market power (*PCM*), higher capital intensity (*CapIntensity*), and are older (*Age*) and larger (*Size*, all *p* < 0.01). The two groups do not differ in the level of tax payments (*CashETR*, *p* = 0.78) and advertising expenses (*Advertising*, *p* = 0.41).

Panel D presents pairwise correlations for the full sample. As I predict, *DivPay* is negatively correlated with *CVTax* (*p* < 0.01). Note that *CVTax* shows a positive correlation with *CVCashFlow*, *CVRoa*, and *CVSales* (all *p* < 0.01). These correlations are all equal to zero after orthogonalizing *CVTax*.

## 4 Main results: Volatility of tax payments and dividend payouts

Table 3 shows the results of logit regressions that examine the decision to pay a dividend in the full sample. Consistent with H1, the coefficient on *CVTax* is negative and significant in column (1) (*p* < 0.01). In column (3), I replace *CVCashFlow*, *CVRoa*, and *CVSales* with *FactorScore*, which is a composite measure of a firm's operating risk. I create this measure by extracting the factor score from a principal component analysis of *CVCashFlow*, *CVRoa*, and *CVSales* (untabulated).^25^ The coefficient on *CVTax* remains negative and significant (*p* < 0.01), corroborating the results in column (1). To gauge the economic magnitude of the estimates, I present marginal effects in columns (2) and (4). The marginal effect of *CVTax* in column (2) suggests that a one standard deviation increase in the volatility of tax payments is associated with a 4.9 percentage point lower probability of dividend payouts. For comparison, a one standard deviation increase in the volatility of operating cash flows (sales) lowers the likelihood of dividend payouts by 3.6 (4.9) percentage points.

**TABLE 3:** Tests of H1: Volatility of tax payments and the probability of dividend payouts

	DV = *DivPay*			
	(1)	(2)	(3)	(4)
	Coeff.	ME	Coeff.	ME
	(SE)		(SE)	
*CVTax*	−0.212***	−0.049	−0.209***	−0.048
	(0.030)		(0.030)	
*TaxAvoidance*	−0.022	−0.005	−0.025	−0.006
	(0.030)		(0.030)	
*CashFlow*	0.128***	0.029	0.124***	0.029
	(0.035)		(0.036)	
*CVCashFlow*	−0.155***	−0.036		
	(0.026)			
*CVRoa*	−0.044***	−0.010		
	(0.017)			
*CVSales*	−0.213***	−0.049		
	(0.031)			
*FactorScore*			−0.278***	−0.064
			(0.029)	
*Cash*	−0.018	−0.004	−0.022	−0.005
	(0.043)		(0.043)	
*MTB*	0.155***	0.036	0.158***	0.036
	(0.043)		(0.043)	
*SalesGrowth*	−0.248***	−0.057	−0.251***	−0.058
	(0.025)		(0.025)	
*AssetGrowth*	−0.248***	−0.057	−0.248***	−0.057
	(0.023)		(0.023)	
*RETE*	0.438***	0.101	0.443***	0.102
	(0.057)		(0.057)	
*Age*	0.687***	0.159	0.690***	0.159
	(0.041)		(0.041)	
*Size*	0.614***	0.142	0.612***	0.141
	(0.048)		(0.048)	
*Leverage*	−0.103***	−0.024	−0.107***	−0.025
	(0.039)		(0.039)	
*PCM*	0.014	0.003	0.007	0.002
	(0.045)		(0.045)	
*Loss*	−0.205***	−0.047	−0.207***	−0.048
	(0.021)		(0.021)	
*NOL*	−0.137***	−0.032	−0.138***	−0.032
	(0.049)		(0.048)	
*R&D*	−0.332***	−0.077	−0.332***	−0.077
	(0.056)		(0.057)	
*SGA*	0.048	0.011	0.053	0.012
	(0.049)		(0.049)	
*Advertising*	−0.039	−0.009	−0.040	−0.009
	(0.041)		(0.040)	
*CapIntensity*	0.289***	0.067	0.293***	0.068
	(0.046)		(0.046)	
Intercept	−0.320***		−0.302***	
	(0.083)		(0.084)	
Year FE	Y		Y	
Industry FE	Y		Y	
Observations	33,023		33,023	
Pseudo *R* ^2^	0.290		0.290	

*Notes*: This table presents regression results for tests of H1 that examine the association between the volatility of tax payments and the probability of dividend payouts for the full sample. Columns (1) and (3) (columns (2) and (4)) report coefficients (marginal effects) for a logit regression based on equation (1). I calculate marginal effects while holding continuous variables at their means. All variables are standardized to have a mean of zero and a standard deviation of one prior to fitting regressions. All regressions are estimated with year and industry fixed effects. I report heteroscedasticity‐robust standard errors clustered by firm in parentheses. Variables are defined in the Appendix. *** represents a significance level of 0.01 (two‐tailed).

Table 4 shows the results of linear regressions that examine dividend levels in the subsample of dividend‐paying firms.^26^ In column (1), *CVTax* is associated with a lower amount of dividend payouts (*p* < 0.01), which is consistent with H2. I find similar results when including *FactorScore* in column (2).^27^ In terms of economic magnitude, a one standard deviation increase in the volatility of tax payments is associated with 3% lower dividend levels (column (1)). For the average dividend‐paying firm, this effect is equivalent to $6.8 million less in dividends paid.^28^ For comparison, a one standard deviation increase in the volatility of operating cash flow (sales) is associated with 7.7% (1.8%) lower dividend levels, which equals $17.4 ($4.1) million less in dividends paid.

**TABLE 4:** Tests of H2: Volatility of tax payments and dividend levels

	DV = *DivAmount*	
	(1)	(2)
	Coeff.	Coeff.
	(SE)	(SE)
*CVTax*	−0.030***	−0.028**
	(0.011)	(0.010)
*TaxAvoidance*	0.018	0.018
	(0.012)	(0.012)
*CashFlow*	0.056***	0.059***
	(0.014)	(0.013)
*CVCashFlow*	−0.077***	
	(0.011)	
*CVRoa*	−0.013	
	(0.009)	
*CVSales*	−0.018*	
	(0.010)	
*FactorScore*		−0.068***
		(0.012)
*Cash*	0.005	0.007
	(0.018)	(0.019)
*MTB*	0.093***	0.093***
	(0.014)	(0.015)
*SalesGrowth*	−0.036***	−0.037***
	(0.008)	(0.009)
*AssetGrowth*	−0.092***	−0.093***
	(0.010)	(0.011)
*RETE*	0.055***	0.057***
	(0.019)	(0.019)
*Age*	0.202***	0.197***
	(0.075)	(0.074)
*Size*	−0.440***	−0.418***
	(0.088)	(0.087)
*Leverage*	−0.087***	−0.086***
	(0.025)	(0.024)
*PCM*	0.063***	0.067***
	(0.021)	(0.021)
*Loss*	−0.021**	−0.020**
	(0.009)	(0.009)
*NOL*	−0.041***	−0.042***
	(0.014)	(0.014)
*R&D*	0.019	0.019
	(0.030)	(0.030)
*SGA*	−0.017	−0.018
	(0.053)	(0.052)
*Advertising*	−0.013	−0.014
	(0.022)	(0.021)
*CapIntensity*	0.041	0.042
	(0.029)	(0.029)
Intercept	−4.160***	−4.154***
	(0.059)	(0.043)
Year FE	Y	Y
Firm FE	Y	Y
Observations	13,612	13,612
Adjusted *R* ^2^	0.169	0.167

*Notes*: This table presents regression results for tests of H2 that examine the association between the volatility of tax payments and the amount of dividend payouts for the subsample. All columns report coefficients for a linear regression based on equation (1). All variables are standardized to have a mean of zero and a standard deviation of one prior to fitting regressions. All regressions are estimated with year and firm fixed effects. I report heteroscedasticity‐robust standard errors clustered by industry in parentheses. Variables are defined in the Appendix. *, **, and *** represent significance levels of 0.10, 0.05, and 0.01, respectively (two‐tailed).

The results in Tables 3 and 4 suggest that the volatility of tax payments has a meaningful effect on dividend payouts that is incremental to the effect of operating risk.^29^ This result is reasonable because tax payments can be a significant cash flow component.^30^ More broadly, my estimates also align with prior studies on the economic consequences of volatile tax payments, such as Guenther et al. (2017) and Saavedra (2019).^31^

The results for the remaining determinants are as expected. The probability of dividend payouts in Table 3 is negatively associated with sales growth (*SalesGrowth*), asset growth (*AssetGrowth*), long‐term debt (*Leverage*), the presence of a loss (*Loss*), the amount of tax loss carryforward (*NOL*), and R&D expenses (*R&D*), and positively associated with cash flow (*CashFlow*), the market‐to‐book ratio (*MTB*), retained earnings (*RETE*), age (*Age*), size (*Size*), and capital intensity (*CapIntensity*). Similarly, dividend levels in Table 4 are negatively related to sales growth (*SalesGrowth*), asset growth (*AssetGrowth*), size (*Size*), long‐term debt (*Leverage*), the presence of a loss (*Loss*), and the amount of tax loss carryforward (*NOL*), and positively related to cash flow (*CashFlow*), the market‐to‐book ratio (*MTB*), retained earnings (*RETE*), age (*Age*), and product market power (*PCM*).

Taken together, the results in this section support H1 and H2: firms with more volatile tax payments are less likely to distribute dividends; and, if payouts are made, their dividends are lower in magnitude. The results also suggest that the effect of the volatility of tax payments on dividend payouts is incremental to operating risk.

## 5 Cross‐sectional evidence

### Agency motives for paying a dividend

As noted, dividends are an important mechanism for shareholders to alleviate agency conflicts (Easterbrook 1984). Shareholders demanding dividends because of agency motives are willing to incur the costs of external financing and may force managers to raise external capital in order to achieve the desired payout policy (Jensen 1986; Farre‐Mensa et al. 2021). Thus, a potentially higher likelihood of liquidity shortfalls due to volatile tax payments should be less relevant for payout decisions dominated by agency motives, suggesting a weaker relation between the volatility of tax payments and the probability of dividend payouts.

I use firms' institutional ownership to identify payout decisions dominated by agency motives. Institutional investors are more adept than retail investors at implementing a payout policy that alleviates agency conflicts (Allen et al. 2000; Crane et al. 2016). I obtain ownership data from the Thomson Reuters Institutional (13f) Holdings Database and classify firms with a mean share of institutional ownership in the top annual quartile as having high institutional ownership (*HighInst*). I estimate the logit regression for each subsample and present the results for firms with low institutional ownership in columns (1) and (2) and for firms with high institutional ownership in columns (3) and (4) of Table 5.^32^ The coefficient on *CVTax* is negative and significant in column (1) (*p* < 0.01), but insignificant in column (3) (*p* = 0.93). The effect in column (3) is significantly weaker (*p* < 0.01, one‐tailed),^33^ consistent with lower agency costs weakening the relation between the volatility of tax payments and the probability of dividend payouts.

**TABLE 5:** Cross‐sectional evidence: Agency motives for paying a dividend

	DV = *DivPay*			
	*HighInst* = 0		*HighInst* = 1	
Subsample	Low Institutional Ownership		High Institutional Ownership	
	(1)	(2)	(3)	(4)
	Coeff.	ME	Coeff.	ME
	(SE)		(SE)	
*CVTax*	−0.219***	−0.052	−0.006	−0.001
	(0.041)		(0.062)	
Controls	Y		Y	
Year FE	Y		Y	
Industry FE	Y		Y	
Observations	16,630		5,535	
Pseudo *R* ^2^	0.333		0.232	
*CVTax* (1) < (3) *p*‐value (one‐tailed)	0.003			

*Notes*: This table presents regression results for a cross‐sectional test based on agency motives for dividend payouts for the full sample. I classify a firm with a mean share of institutional ownership in the top annual quartile as having high institutional ownership. I obtain ownership data from the Thomson Reuters Institutional (13f) Holdings Database. Columns (1) and (3) (columns (2) and (4)) report coefficients (marginal effects) for a logit regression based on equation (1). I calculate marginal effects while holding continuous variables at their means. All variables are standardized to have a mean of zero and a standard deviation of one in each subsample prior to fitting regressions. All regressions are estimated with year and industry fixed effects. I report heteroscedasticity‐robust standard errors clustered by firm in parentheses. Variables are defined in the Appendix. *** represents a significance level of 0.01 (two‐tailed).

### High UTB balances

I next test whether the association between the volatility of tax payments and the amount of dividend payouts varies with a firm's UTB reserves. Hanlon et al. (2017) show that firms with higher UTB balances tend to sideline more cash for tax purposes. If firms hold cash for tax reasons, volatile tax payments might not increase the likelihood of liquidity shortfalls (Hanlon et al. 2017), making it easier for these firms to maintain consistent dividend levels over time. Based on this argument, I expect a weaker relation between the volatility of tax payments and the dividend levels of firms reporting high UTB reserves.^34^

In Table 6, I estimate the linear regression for firms with low UTB reserves in column (1) and for firms with high UTB reserves in column (2).^35^ The coefficient on *CVTax* is negative and significant in column (1) (*p* = 0.02), but insignificant in column (2) (*p* = 0.91). As predicted, the effect of *CVTax* is significantly weaker in column (2) (*p* = 0.08, one‐tailed).^36^ These results suggest that the relation between the volatility of tax payments and dividend levels is weaker for firms reporting higher UTB balances, consistent with the idea that sidelining more cash for tax reasons makes firms less sensitive to volatile tax payments in their dividend payout decisions.

**TABLE 6:** Cross‐sectional evidence: High UTB balances

	DV = *DivAmount*	
	*HighUTB* = 0	*HighUTB* = 1
Subsample	Low UTBs	High UTBs
	(1)	(2)
	Coeff.	Coeff.
	(SE)	(SE)
*CVTax*	−0.033**	0.003
	(0.014)	(0.022)
Controls	Y	Y
Year FE	Y	Y
Firm FE	Y	Y
Observations	3,457	1,114
Adjusted *R* ^2^	0.120	0.178
*CVTax* (1) < (2) *p*‐value (one‐tailed)	0.079	

*Notes*: This table presents regression results for a cross‐sectional test based on a firm's UTB balances for the subsample. I classify a firm with a year‐end UTB balance (scaled by total assets) in the top annual quartile as reporting high UTB reserves. All columns report coefficients for a linear regression based on equation (1). All variables are standardized to have a mean of zero and a standard deviation of one in each subsample prior to fitting regressions. All regressions are estimated with year and firm fixed effects. I report heteroscedasticity‐robust standard errors clustered by industry in parentheses. Variables are defined in the Appendix. ** represents a significance level of 0.05 (two‐tailed).

### Additional tests: Borrowing costs and costs of dividend reductions

I conduct two additional tests to provide empirical support for the arguments underlying H1 and H2. First, since firms that experience a liquidity shortfall might require external debt to finance dividend payouts (Farre‐Mensa et al. 2021), I expect a stronger relation between volatile tax payments and the decision to pay a dividend for firms with high borrowing costs. To test this conjecture, I split the full sample based on whether a firm has an S&P Issuer Credit Rating or not (*Rating*). Unrated firms have limited access to public debt markets and therefore face high borrowing costs (Whited 1992; Faulkender and Petersen 2006). I estimate the logit regression for rated firms in columns (1) and (2) and for unrated firms in columns (3) and (4) of Table S2 in supporting information in the online Appendix. Although the coefficient on *CVTax* is negative and significant in both subsamples (all *p* < 0.01), the effect is stronger in column (3) (*p* = 0.04, one‐tailed). These results suggest that the association between volatile tax payments and the probability of dividend payouts is stronger for firms with high borrowing costs, which is consistent with the theoretical arguments for H1.

Second, I expect a weaker relation between the volatility of tax payments and the dividend levels of firms with relatively high growth. These firms incur low costs for dividend reductions and have flexibility in adapting their dividend payouts (Leary and Michaely 2011). I follow DeAngelo et al. (2006) and identify a growing firm as having a ratio of retained earnings to shareholder equity in the bottom annual quartile (*LowRETE*).^37^ In Table S3 in supporting information in the online Appendix, I estimate the linear regression for growing firms in column (1) and for more mature firms in column (2). The coefficient on *CVTax* is insignificant in column (1) (*p* = 0.48), but negative and significant in column (2) (*p* = 0.05). The effect of *CVTax* in column (1) is significantly weaker (*p* = 0.02, one‐tailed), consistent with the costs of dividend reductions moderating the effect of volatile tax payments on dividend levels.

## 6 Supplementary analysis

### Sensitivity analysis and robustness tests

#### Alternative measures of the volatility of tax payments

As a first sensitivity analysis, I use alternative measures of the volatility of tax payments and present results for the full sample (the subsample) in panel A (B) of Table 7.^38^ First, I reestimate the main analysis but require positive pre‐tax income to calculate annual cash ETRs. This approach excludes loss observations from the sample. The coefficients on *CVTax* remain negative and significant in column (1) (both *p* < 0.09); the effect sizes are slightly smaller than in the main tests. These results suggest that loss firms do not drive my inferences.^39^

**TABLE 7:** Alternative measures of the volatility of tax payments

**Panel A:** Volatility of tax payments and the probability of dividend payouts				
	DV = *DivPay*			
	(1)	(2)	(3)	(4)
	ME	ME	ME	ME
	(SE)	(SE)	(SE)	(SE)
*CVTax*	−0.040***			
	(0.009)			
*SDTax*		−0.017***		
		(0.006)		
*HighTaxVol*			−0.026***	
			(0.009)	
*GAAPCVTax*				−0.047***
				(0.007)
*TaxAvoidance*	−0.067***	−0.032***	−0.075***	
	(0.011)	(0.006)	(0.012)	
*TaxAvoidanceGAAP*				0.002
				(0.006)
*CashFlow*	0.014	0.046***	0.013	0.033***
	(0.011)	(0.008)	(0.012)	(0.008)
*CVCashFlow*	−0.019**		−0.041***	−0.034***
	(0.008)		(0.011)	(0.006)
*CVRoa*	−0.014***		−0.018***	−0.010***
	(0.006)		(0.007)	(0.004)
*CVSales*	−0.043***		−0.034***	−0.053***
	(0.009)		(0.009)	(0.007)
*SDCashFlow*		−0.056***		
		(0.009)		
*SDRoa*		−0.037***		
		(0.007)		
*SDSales*		−0.016**		
		(0.008)		
*Cash*	−0.005	−0.006	−0.008	−0.002
	(0.013)	(0.010)	(0.013)	(0.010)
*MTB*	0.038***	0.040***	0.033**	0.033***
	(0.013)	(0.010)	(0.013)	(0.009)
*SalesGrowth*	−0.063***	−0.059***	−0.060***	−0.061***
	(0.008)	(0.006)	(0.007)	(0.006)
*AssetGrowth*	−0.060***	−0.054***	−0.051***	−0.058***
	(0.006)	(0.005)	(0.006)	(0.005)
*RETE*	0.032**	0.107***	0.047***	0.106***
	(0.013)	(0.013)	(0.014)	(0.014)
*Age*	0.191***	0.154***	0.183***	0.153***
	(0.012)	(0.009)	(0.012)	(0.009)
*Size*	0.132***	0.142***	0.126***	0.148***
	(0.014)	(0.011)	(0.014)	(0.011)
*Leverage*	−0.012	−0.030***	−0.025**	−0.026***
	(0.012)	(0.009)	(0.012)	(0.009)
*PCM*	0.010	−0.002	0.011	0.001
	(0.013)	(0.010)	(0.014)	(0.010)
*Loss*	−0.012**	−0.043***	−0.026***	−0.037***
	(0.005)	(0.005)	(0.005)	(0.005)
*NOL*	−0.013	−0.033***	−0.009	−0.030**
	(0.012)	(0.011)	(0.011)	(0.012)
*R&D*	−0.048***	−0.079***	−0.055***	−0.080***
	(0.015)	(0.013)	(0.015)	(0.013)
*SGA*	−0.001	0.018	0.010	0.012
	(0.015)	(0.011)	(0.015)	(0.011)
*Advertising*	−0.010	−0.008	−0.011	−0.008
	(0.012)	(0.009)	(0.013)	(0.009)
*CapIntensity*	0.088***	0.064***	0.091***	0.063***
	(0.014)	(0.010)	(0.015)	(0.010)
Intercept	0.000	0.000	0.000	0.000
	(0.000)	(0.000)	(0.000)	(0.000)
Year FE	Y	Y	Y	Y
Industry FE	Y	Y	Y	Y
Observations	19,152	33,023	18,013	35,340
Pseudo *R* ^2^	0.261	0.288	0.250	0.300

**Panel B:** Volatility of tax payments and the amount of dividend payouts				
	DV = *DivAmount*			
	(1)	(2)	(3)	(4)
	Coeff.	Coeff.	Coeff.	Coeff.
	(SE)	(SE)	(SE)	(SE)
*CVTax*	−0.020*			
	(0.012)			
*SDTax*		−0.021**		
		(0.009)		
*HighTaxVol*			−0.027**	
			(0.010)	
*GAAPCVTax*				−0.035***
				(0.011)
*TaxAvoidance*	−0.010	0.009	−0.032**	
	(0.014)	(0.013)	(0.016)	
*TaxAvoidanceGAAP*				0.002
				(0.013)
*CashFlow*	0.050***	0.068***	0.056***	0.055***
	(0.013)	(0.013)	(0.015)	(0.013)
*CVCashFlow*	−0.063***		−0.072***	−0.080***
	(0.013)		(0.013)	(0.011)
*CVRoa*	−0.011		−0.004	−0.015
	(0.008)		(0.012)	(0.010)
*CVSales*	−0.012		−0.022**	−0.021*
	(0.010)		(0.011)	(0.011)
*SDCashFlow*		−0.025*		
		(0.013)		
*SDRoa*		0.004		
		(0.011)		
*SDSales*		−0.035***		
		(0.010)		
*Cash*	−0.004	0.004	−0.009	0.000
	(0.019)	(0.019)	(0.019)	(0.019)
*MTB*	0.107***	0.094***	0.105***	0.104***
	(0.014)	(0.015)	(0.016)	(0.016)
*SalesGrowth*	−0.036***	−0.040***	−0.033***	−0.038***
	(0.012)	(0.009)	(0.008)	(0.009)
*AssetGrowth*	−0.097***	−0.092***	−0.099***	−0.097***
	(0.012)	(0.011)	(0.013)	(0.010)
*RETE*	0.002	0.062***	0.019	0.052***
	(0.019)	(0.020)	(0.018)	(0.019)
*Age*	0.199***	0.194**	0.222**	0.212***
	(0.067)	(0.075)	(0.089)	(0.077)
*Size*	−0.479***	−0.398***	−0.366***	−0.448***
	(0.103)	(0.086)	(0.111)	(0.083)
*Leverage*	−0.064***	−0.089***	−0.066***	−0.082***
	(0.024)	(0.025)	(0.025)	(0.025)
*PCM*	0.047**	0.070***	0.039	0.067***
	(0.021)	(0.023)	(0.026)	(0.023)
*Loss*	0.001	−0.026**	−0.006	−0.023**
	(0.006)	(0.010)	(0.009)	(0.009)
*NOL*	−0.021	−0.047***	−0.044***	−0.044***
	(0.018)	(0.015)	(0.016)	(0.012)
*R&D*	0.010	0.020	0.011	0.018
	(0.034)	(0.031)	(0.037)	(0.030)
*SGA*	−0.054	−0.013	−0.042	−0.021
	(0.056)	(0.053)	(0.061)	(0.053)
*Advertising*	−0.004	−0.014	0.002	−0.008
	(0.025)	(0.021)	(0.030)	(0.021)
*CapIntensity*	0.098***	0.046	0.100***	0.057*
	(0.032)	(0.028)	(0.035)	(0.032)
Intercept	−4.141***	−4.151***	−4.148***	−4.172***
	(0.055)	(0.059)	(0.051)	(0.059)
Year FE	Y	Y	Y	Y
Firm FE	Y	Y	Y	Y
Observations	10,142	13,612	9,346	13,922
Adjusted *R* ^2^	0.193	0.163	0.183	0.161

*Notes*: This table presents regression results for tests using alternative measures of the volatility of tax payments. Panel A (B) includes samples of dividend‐paying and non‐dividend‐paying firms (dividend‐paying firms). Panel A (B) reports marginal effects for a logit regression (coefficients for a linear regression) based on equation (1). I calculate marginal effects while holding continuous variables at their means. All variables are standardized to have a mean of zero and a standard deviation of one prior to fitting regressions. In panel A (B), all regressions are estimated with year and industry fixed effects (year and firm fixed effects). In panel A (B), I report heteroscedasticity‐robust standard errors clustered by firm (industry) in parentheses. Variables are defined in the Appendix. *, **, and *** represent significance levels of 0.10, 0.05, and 0.01, respectively (two‐tailed).

Second, I follow Guenther et al. (2017) and Saavedra (2019) and replace *CVTax* with the standard deviation of annual cash ETRs (*SDTax*).^40^ I similarly recalculate the three proxies for operating risk (*SDCashFlow*, *SDRoa*, and *SDSales*). In column (2), the coefficients on *SDTax* are negative and significant (both *p* < 0.02), corroborating the robustness of my findings. The effect sizes of *SDTax* are slightly smaller than in the main tests but continue to suggest that the volatility of tax payments is an economically important determinant of dividend payouts.^41^

Third, I use the measure developed by Saavedra (2019), in which an unusually large tax payment serves as an indicator for volatile tax payments. I classify a firm with an annual cash ETR and an average annual cash ETR larger than 0.6 in any of the years *t* − 4 to *t* as exhibiting a high volatility of tax payments (*HighTaxVol*). Consistent with my main results, the coefficients on *HighTaxVol* are negative and significant in column (3) (both *p* < 0.02).

Finally, I recalculate my primary measure using annual GAAP instead of cash ETRs (*GAAPCVTax*). Consistent with a cash ETR measure, volatility stemming from a firm's tax strategy as measured by annual GAAP ETRs is negatively associated with the probability of dividend payouts and their amount (column (4), both *p* < 0.01).

#### Controlling for operating risk and differences in firm characteristics

As noted, operating risk could drive the relation between *CVTax* and dividend payouts. Although I orthogonalize *CVTax* with respect to several proxies for operating risk in the main analysis and find that the volatility of tax payments has an incremental effect, this section presents alternative approaches to alleviate remaining concerns about operating risk and differences in firm characteristics driving my results. Table S4 in supporting information in the online Appendix presents the results.^42^

As a first approach, I create a measure that captures a firm's volatility of tax payments independent of its operating risk. Specifically, I sort observations into deciles of volatility of tax payments (*RCVTax*) within deciles of volatility of operating cash flows (*RCVCashFlow*). As a result, a firm with a high volatility of tax payments but low operating risk is classified the same as a firm with a high volatility of tax payments and high operating risk. Consistent with my main results, the coefficients on *RCVTax* in columns (1) and (4) are negative and significant for the full sample and the subsample, respectively (both *p* < 0.01). The effect sizes of *RCVTax* and *RCVCashFlow* are slightly larger than in the main tests.

A related concern is that differences in firm characteristics could drive my results. To alleviate this concern and also to address concerns about functional form (Hainmueller 2012), I match dividend‐paying firms with non‐dividend‐paying firms on all determinants in equation (1), except for *CVTax*. This approach matches firms with similar characteristics but different dividend‐paying status and different levels of volatility in their tax payments. I first use entropy balancing.^43^ The coefficient on *CVTax* in column (2) remains negative and significant (*p* < 0.01), and the marginal effect is similar to the main tests. The coefficients on *CVCashFlow*, *CVRoa*, and *CVSales* are all insignificant (all *p* > 0.77), consistent with entropy balancing adjusting for differences in firm characteristics. I next use propensity score matching.^44^ The coefficient on *CVTax* in column (3) is again negative and significant (*p* < 0.01). The marginal effect is slightly larger than in the main tests, but the coefficients on *CVCashFlow*, *CVRoa*, and *CVSales* are again insignificant (all *p* > 0.24). Collectively, the results in this section provide additional support that the negative effect of volatile tax payments on dividend payouts is incremental to operating risk. The results also suggest that differences in firm characteristics are unlikely to affect my results.

#### Changes specification

Another concern is that time‐invariant firm characteristics or reverse causality could drive my findings. Although firm fixed effects alleviate this concern in the tests of dividend levels, I use a changes specification also to address it in the tests of the probability of dividend payouts (Lancaster 2000). Specifically, I estimate a multinomial logistic regression to examine the effect of changes in the volatility of tax payments (and in control variables) on changes in dividend payouts, representing dividend omissions or initiations.^45^ Table S7 in supporting information in the online Appendix presents the results.

In column (1), the positive and significant coefficient on Δ *CVTax* indicates that an increase in the volatility of tax payments raises the probability of dividend omissions (*p* < 0.01). For dividend initiations in column (2), the coefficient on Δ *CVTax* is negative as expected but marginally insignificant (*p* = 0.19). Thus, changes in the volatility of tax payments affect the likelihood of dividend omissions while having a weaker effect on dividend initiations. In column (3), I estimate a changes specification also for the tests of dividend levels.^46^ Consistent with my main results, the negative coefficient on Δ *CVTax* (*p* = 0.05) suggests that an increase (decrease) in the volatility of tax payments is associated with lower (higher) dividend levels. In sum, these results alleviate endogeneity concerns and thus corroborate the robustness of my findings.

#### Volatility of tax payments and variation in future after‐tax cash flows

The hypothesized relation between the volatility of tax payments and dividend payouts rests on the assumption that managers can form expectations about the cash flow implications of volatile tax payments and use this information in a dividend payout decision. To support this argument, I examine the relation between *CVTax* and the volatility of future after‐tax cash flows. I calculate *CVTotalCF* as the coefficient of variation for annual net operating cash flows (scaled by lagged total assets) over the five‐year period *t* + 1 to *t* + 5. I then follow Drake et al. (2019) and regress *CVTotalCF* on current measures of *CVTax* and *TaxAvoidance* as well as of *CashFlow*, *CVCashFlow*, *CVRoa*, and *CVSales*. Table 8 presents the results.^47^

**TABLE 8:** Volatility of tax payments and variation in future after‐tax cash flow

	DV = *CVTotalCF*	
	(1)	(2)
	Coeff.	Coeff.
	(SE)	(SE)
*CVTax*	0.058***	0.043**
	(0.019)	(0.019)
*TaxAvoidance*	−0.082***	−0.065***
	(0.025)	(0.025)
*CashFlow*		−0.067***
		(0.019)
*CVCashFlow*		0.090***
		(0.027)
*CVRoa*		−0.009
		(0.019)
*CVSales*		−0.054***
		(0.020)
Intercept	0.432***	0.416***
	(0.079)	(0.072)
Year FE	Y	Y
Industry FE	Y	Y
Observations	18,171	18,171
Adjusted *R* ^2^	0.003	0.007

*Notes*: This table presents regression results for tests that examine the association between volatile tax payments and variation in future after‐tax cash flows for the full sample. All columns report coefficients for a linear regression. All variables are standardized to have a mean of zero and a standard deviation of one prior to fitting regressions. All regressions are estimated with year and industry fixed effects. I report heteroscedasticity‐robust standard errors clustered by firm in parentheses. Variables are defined in the Appendix. ** and *** represent significance levels of 0.05 and 0.01, respectively (two‐tailed).

In column (1), the coefficient on *CVTax* is positive and significant (*p* < 0.01). I find similar results when including the full set of variables in column (2).^48^ Taken together, these results suggest that the current volatility of tax payments could be informative to managers about the potential future cash flow implications of a firm's tax strategy. Yet, given the low explanatory power of the regression models in Table 8, I note that some degree of caution is appropriate when drawing this conclusion.

### Volatility of tax payments as a distinct signal for dividend payouts

My results so far suggest that the volatility of tax payments has an incremental effect on dividend payouts, but the direction of the effect coincides with the effect of operating cash flow volatility. In this section, I test whether the volatility of tax payments can be informative about a firm's dividend payouts in cases where operating cash flow volatility provides no signal. I sort observations into annual quartiles of *CVCashFlow* and examine the effects of *CVTax* and *CVCashFlow* separately for each quartile. Table 9 presents the results.

**TABLE 9:** Volatility of tax payments and dividend payouts by quartiles of operating cash flow volatility

**Panel A:** Volatility of tax payments and the probability of dividend payouts				
	DV = *DivPay*			
Subsample	Quartile 1	Quartile 2	Quartile 3	Quartile 4
	(1)	(2)	(3)	(4)
	ME	ME	ME	ME
	(SE)	(SE)	(SE)	(SE)
*CVTax*	−0.047***	−0.044***	−0.030***	−0.021***
	(0.014)	(0.012)	(0.010)	(0.007)
*CVCashFlow*	0.069***	0.011	−0.009	−0.025***
	(0.020)	(0.009)	(0.008)	(0.007)
Additional controls	Y	Y	Y	Y
Year FE	Y	Y	Y	Y
Industry FE	Y	Y	Y	Y
Observations	8,261	8,255	8,258	8,249
Pseudo *R* ^2^	0.338	0.251	0.224	0.222
*CVTax* = *CVCashFlow p*‐value (two‐tailed)	<0.001	<0.001	0.076	0.585

**Panel B:** Volatility of tax payments and the amount of dividend payouts				
	DV = *DivAmount*			
Subsample	Quartile 1	Quartile 2	Quartile 3	Quartile 4
	(1)	(2)	(3)	(4)
	Coeff.	Coeff.	Coeff.	Coeff.
	(SE)	(SE)	(SE)	(SE)
*CVTax*	−0.055*	−0.051**	−0.023	−0.015
	(0.029)	(0.020)	(0.025)	(0.023)
*CVCashFlow*	−0.032	−0.038*	−0.013	−0.083***
	(0.022)	(0.020)	(0.022)	(0.025)
Additional controls	Y	Y	Y	Y
Year FE	Y	Y	Y	Y
Industry FE	Y	Y	Y	Y
Observations	3,408	3,402	3,403	3,399
Adjusted *R* ^2^	0.379	0.384	0.315	0.332
*CVTax* = *CVCashFlow p*‐value (two‐tailed)	0.552	0.636	0.782	0.034

*Notes*: This table presents regression results for tests that examine the relation between the volatility of tax payments and dividend payouts by quartiles of operating cash flow volatility. Panel A (B) includes tests based on the full sample (the subsample). Panel A (B) reports marginal effects for a logit regression (coefficients for a linear regression) based on equation (1). In both panels, I estimate regressions by annual quartiles of *CVCashFlow*. I calculate marginal effects while holding continuous variables at their means. All variables are standardized to have a mean of zero and a standard deviation of one prior to fitting regressions. All regressions are estimated with year and industry fixed effects. I report heteroscedasticity‐robust standard errors clustered by firm in parentheses. Variables are defined in the Appendix. *, **, and *** represent significance levels of 0.10, 0.05, and 0.01, respectively (two‐tailed).

In the full sample (panel A), the coefficients on *CVTax* are negative and significant for all four quartiles of cash flow risk (all *p* < 0.01). The coefficients on *CVCashFlow*, in contrast, are negative and significant only in column (4) (*p* < 0.01) and even positive and significant in column (1) (*p* < 0.01). *F*‐tests suggest that the coefficients on *CVTax* and *CVCashFlow* differ significantly in columns (1) to (3) (all *p* < 0.08). These results suggest that, for firms with low cash flow risk, the volatility of tax payments can provide a distinct negative signal for the probability of dividend payouts. Results for the subsample in panel B are similar but less pronounced. The coefficients on *CVTax* (*CVCashFlow*) are negative and significant only in columns (1) and (2) (columns (2) and (4)) (all *p* < 0.06).^49^ The coefficients on *CVTax* and *CVCashFlow* do not significantly differ in columns (1) to (3) (all *p* > 0.55). Overall, these results suggest that the volatility of tax payments can be incrementally informative about dividend payouts, especially for the probability of dividend payouts of firms with low cash flow risk.

### Volatility of tax payments and future dividend payouts

I next examine whether the current volatility of tax payments is associated with future dividend payouts. Since dividend payouts tend to be sticky over time (Lintner 1956; Brav et al. 2005), I use a firm's current payouts to predict its dividends in the future. Specifically, I regress *DivPay* and *DivAmount* measured for each of the years from *t* + 1 to *t* + 5 on their values in year *t*. By including *CVTax* as an additional predictor, I can test whether the current volatility of tax payments is incrementally informative about a firm's future dividend payouts over its current dividend status. As expected, the results in both panels of Table S8 in supporting information in the online Appendix suggest that current dividend payouts are strong predictors of future payouts. Importantly, the coefficients on *CVTax* are all negative and significant (all *p* < 0.01). These results suggest that the current volatility of tax payments is associated with future dividend payouts, which is consistent with the idea that the current volatility of tax payments can be incrementally useful in predicting future payouts.

### Volatility of tax payments and alternative distribution channels

Instead of paying a dividend, firms could use other channels to distribute funds to their shareholders. Share repurchases are prevalent among US firms (Skinner 2008) because these payouts do not create expectations among investors and are therefore more flexible than regular dividends (Jagannathan et al. 2000). Thus, firms with more volatile tax payments might rely more strongly on this distribution channel. I examine this conjecture in supporting information in the online Appendix (Table S9). *CVTax* is highest for firms using neither channel (panel A) and lowest for firms that distribute dividends and repurchase shares (panel D).^50^ Moreover, the mean of *CVTax* for share‐repurchasing firms (panel B) is larger than for firms that only distribute dividends (panel C, *p* < 0.01). In Table S10 in supporting information in the online Appendix, I repeat this analysis for special dividends as another flexible form of payout.^51^ *CVTax* is again highest for firms using neither distribution channel (panel A) and lowest for firms using both (panel D). The mean of *CVTax* in panel B is larger than in panel C (*p* < 0.01). Overall, these results suggest that, instead of paying a regular dividend, firms with more volatile tax payments tend to prefer more flexible distribution channels, such as share repurchases or special dividends.

## 7 Conclusion

This study examines the payout consequences of volatile tax payments. I predict and find that firms with more volatile tax payments are less likely to pay a dividend and their dividends are lower in magnitude when doing so. The effect of volatile tax payments on dividend payouts is economically meaningful and incremental to a firm's operating risk. Specifically, a one standard deviation increase in the volatility of tax payments is associated with a 4.9 percentage point lower probability of dividend payouts and, for the average dividend‐paying firm, a $6.8 million lower amount of dividends paid. Cross‐sectional tests suggest that the relationship between volatile tax payments and the probability of dividend payouts varies with the importance of agency motives for paying a dividend. The association between volatile tax payments and dividend levels is weaker for firms that hold more cash for tax reasons. Moreover, I find that the volatility of tax payments can be incrementally informative over operating cash flow volatility, especially about dividend payouts of firms with low cash flow risk.

My evidence contributes to our understanding of the determinants of dividend payouts by documenting a meaningful effect of the volatility of tax payments that is incremental to operating risk. More volatile tax payments could also signal lower dividend payouts, which is an important aspect in investment strategies. Finally, my study speaks to shareholders by identifying a factor that can curtail dividend payouts as a mechanism to alleviate agency conflicts.

## Supporting information


**Online Appendix.** Supporting information

## Data Availability

Data used in this study are available from public sources identified in the paper.

## Footnotes

1 In addition to orthogonalizing the measure of volatile tax payments in my primary analysis, the tests in section 6 provide corroborating evidence that operating risk is unlikely to drive my findings.

2 Farre‐Mensa et al. (2021) find that firms use external debt to finance dividend payouts if internal funds are not sufficient.

3 The disputed amounts can be substantial. In 2006, GlaxoSmithKline Inc. settled a transfer pricing dispute with the IRS. As the largest dispute in the history of the IRS, the settlement for fiscal years 1989–2005 resulted in tax repayments of $3.4 billion and an abandoned claim of $1.8 billion (IRS 2006). In a similar case, Canadian tax authorities claimed tax repayments of $2.1 billion from Cameco Corp. (Livesey 2016).

4 More precisely, Hutchens and Rego (2015, 1) study the relation between tax risk and overall firm risk. They define tax risk broadly as “all tax‐related uncertainties that surround a firm's transactions, operations, financial reporting decisions, and corporate reputation.”

5 Since I am interested in all sources of variation in tax payments associated with a firm's tax strategy, I focus on the volatility of tax payments rather than on the constructs of tax risk and tax uncertainty.

6 Detailed variable definitions are provided in the Appendix. I set annual cash ETRs to one in case of a positive numerator and a negative denominator (tax payments in a loss year) and to zero in case of a negative numerator and a negative denominator (tax refund in a loss year). I keep loss firms in the sample to avoid a data truncation bias (Henry and Sansing 2018) and because losses are an important determinant of dividend payouts (DeAngelo et al. 1992). I find consistent results when dropping firms from the sample that report (i) a loss (Table 7) or (ii) a tax refund in one of the years between *t* − 4 and *t* (untabulated).

7 As a result, the coefficient of variation is unaffected by the level of cash ETRs. In Table 7, I find consistent results when using the standard deviation of annual cash ETRs (Guenther et al. 2017; Saavedra 2019; Drake et al. 2019).

8 I define *LossFirm* based on pre‐tax income, which is the denominator for annual cash ETRs.

9 For linear models, the Frisch Waugh theorem implies that controlling for correlated covariates removes their effect from the coefficient of interest. As outlined below, I use a nonlinear logit model to examine the probability of dividend payouts. Because this model produces residuals for a categorical response variable (e.g., whether or not to pay a dividend) and not for the underlying continuous latent response, the Frisch Waugh theorem does not apply. As a result, controlling for *CVCashFlow*, *CVRoa*, *CVSales*, and *LossFirm* in the logit regression might not fully remove the effect of operating risk and losses from *CVTax*, potentially overstating the effect size for *CVTax* (Abbott and Carroll 1984). Orthogonalizing *CVTax* prior to estimating the payout regression addresses this issue. I find consistent results (untabulated) when extending the orthogonalization procedure to all control variables in equation (1) and when estimating the payout regression using a linear probability model. Results are also qualitatively similar when using nonorthogonalized values of *CVTax* (untabulated).

10 Results are unchanged (i) when requiring at least 15 observations per industry‐year, in line with Badertscher et al. (2019), and (ii) when not constraining the orthogonalization procedure to industry‐year combinations (untabulated). To implement the orthogonalization procedure and additionally address concerns that correlated covariates might influence the effect sizes of the respective variables, I estimate the Stata command *orthog* and use the output in the subsequent payout regressions.

11 Panel A of Table S1 in the supporting information in the online Appendix shows the results of orthogonalizing *CVTax* on the full sample. For consistency, I use orthogonalized values of *CVTax* also in tests of dividend levels, and present the results for the subsample in panel B. Mean and median values for the coefficients on *CVCashFlow*, *CVRoa*, *CVSales*, and *LossFirm* are positive in both panels. These results indicate that operating risk and financial statement losses are positively associated with the volatility of tax payments. In untabulated tests, I rerun the test in column (1) of Table 4 with values of *CVTax* orthogonalized on the full sample (again by industry‐year) and find consistent results.

12 Results (untabulated) are consistent when rerunning my main tests using a Cragg (1971) hurdle model, which allows managers to simultaneously decide on paying a dividend and on setting the dividend level (Wooldridge 2010, 690).

13 The tests of dividend levels are not subject to sample selection concerns because payouts of zero are a corner solution for the payout decision of non‐dividend‐paying firms (Wooldridge 2010, 804).

14 I use the logarithm, as the variable is positively skewed. My main results are similar without using the logarithm (untabulated).

15 I calculate *CVRoa* based on pre‐tax income to capture variation in operating and nonoperating income. My main results are consistent when calculating *CVRoa* based on earnings before interest and taxes (untabulated).

16 These variables also control for economic risk associated with a firm's tax strategy (Neuman et al. 2020).

17 For instance, a firm's intended dividend policy could influence its general business strategy, which, in turn, might affect the volatility of tax payments (Venkatesh 1989; Sant and Cowan 1994). Moreover, Higgins et al. (2015) show that firms align their tax strategy with their general business strategy.

18 In the supporting information in the online Appendix (Table S7), I use a changes specification as an approach that controls for time‐invariant firm characteristics in logit regressions but that is less prone to the incidental parameters problem (Lancaster 2000) and find consistent results.

19 Industry fixed effects are based on the Fama and French 17 industry classification, available on Kenneth French's website (http://mba.tuck.dartmouth.edu/pages/faculty/ken.french/Data_Library/det_17_ind_port.html). I use Fama and French 17 industry codes because *DivPay* exhibits limited variation in some industries. Thus, estimating a logit regression with industry fixed effects based on a narrower industry definition would lead to a loss in sample size. My results are unchanged when using Fama and French 48 or 2‐digit SIC codes (untabulated).

20 I use distinct clustering strategies to avoid clustering standard errors at the same dimension as the fixed effect included in the regression model. To ensure a sufficient number of clusters (Cameron and Miller 2015), I define industry clusters based on Fama and French 48 industry codes. My results are consistent when using Fama and French 17 industry codes (untabulated).

21 When examining subsamples of the initial samples, I standardize independent variables within each subsample.

22 I take 1993 as the starting date to ensure consistent accounting rules for income taxes. ASC 740 (SFAS No. 109), which changed the accounting for income taxes, became effective for fiscal years starting after December 15, 1992. Although ASC 740 did not alter the calculation of cash taxes paid, prior research suggests that firms strategically respond to legal changes (Scholes et al. 1992; Guenther 1994; Maydew 1997). Such behavior could affect annual cash ETRs as a basis for *CVTax*.

23 Dropping firms incorporated outside the United States is a simple way of excluding foreign firms. However, I acknowledge that differentiating between domestic and foreign firms could be more complex. For example, a firm could be incorporated abroad while its shares are traded on a US stock exchange. By relying on the US incorporation criterion, I remove these firms, consistent with Dyreng et al. (2008).

24 Unless indicated otherwise, all *p*‐values reported in the manuscript are from two‐tailed tests.

25
*CVCashFlow*, *CVRoa*, and *CVSales* load on a single factor with an eigenvalue of 1.18. This factor accounts for 39.4% of the variance. Factor loadings are positive and equal to 0.65 (*CVCashFlow*), 0.34 (*CVRoa*), and 0.67 (*CVSales*), respectively. The correlations between *FactorScore* and the three operating risk proxies are also positive and equal to 0.68 (*CVCashFlow*), 0.37 (*CVRoa*), and 0.75 (*CVSales*), respectively, consistent with the idea that higher values of *FactorScore* indicate greater operating risk.

26 Due to the lack of variation in *LossFirm* for some industry‐years, 82 observations are dropped when orthogonalizing *CVTax* for the subsample, explaining the slight loss in sample size. Inferences are unchanged when replacing *CVTax* with zero for these observations, which is the industry‐year mean of *CVTax* after orthogonalization (untabulated).

27 In the subsample, *CVCashFlow*, *CVRoa*, and *CVSales* load on a single factor with an eigenvalue of 1.38, which accounts for 46.2% of the variance. Factor loadings are positive and amount to 0.66 (*CVCashFlow*), 0.38 (*CVRoa*), and 0.64 (*CVSales*), respectively. The correlations between *FactorScore* and the operating risk variables are positive and equal to 0.77 (*CVCashFlow*), 0.43 (*CVRoa*), and 0.77 (*CVSales*), respectively, again consistent with the idea that higher values of *FactorScore* indicate greater operating risk.

28 I calculate this effect using the mean dividend payouts (2.7% of total assets, panel B of Table 2) and the mean total assets for the subsample of dividend‐paying firms ($8,376 million, untabulated): **−**0.03 × 0.027 × 8,376 = **−**6.8.

29 One explanation for the small effect sizes of *CVRoa*, one proxy for operating risk, is that the measure is based on pre‐tax income, capturing the volatility of operating and nonoperating income. Due to its transitory nature, non‐operating income is primarily used for share repurchases (Jagannathan et al. 2000), which should attenuate the effect of *CVRoa* on dividend payouts. Indeed, when separately controlling for the two dimensions of income volatility, the effect sizes of the volatility of operating income are comparable to those of the other operating risk proxies (untabulated).

30 Accordingly, untabulated descriptive statistics suggest that tax payments, in absolute terms, amount to 35.7% (34.8%) of net operating cash flows for the firms in the full sample (subsample).

31 Guenther et al. (2017) find that the effect of cash ETR volatility on overall firm risk falls between the effects of the volatility of pre‐tax income and the volatility of operating cash flows. Saavedra (2019) shows that the effect of tax volatility on loan spreads is comparable to the effect of the volatility of pre‐tax cash flows. Thus, my finding that volatile tax payments have an effect on dividend payouts that is broadly in the range of the effect of operating risk is consistent with these prior studies. I note, however, that Guenther et al. (2017) and Saavedra (2019) measure their variable of interest using the standard deviation (and not the coefficient of variation) of annual cash ETRs. I apply this approach in Table 7 and find consistent results.

32 For the cross‐sectional tests, I orthogonalize *CVTax* before splitting the respective sample into subsamples.

33 I use a fully interacted model to test for differences in the coefficients on *CVTax* in Table 5 and Table S2 in supporting information in the online Appendix (Allison 1999).

34 I note that higher UTB balances ex ante may also be indicative of greater technical or legal tax risk (Lisowsky et al. 2013), suggesting a stronger association between the volatility of tax payments and dividend levels. To support a cash‐based explanation, I replicate the main analysis in Hanlon et al. (2017) and find that higher UTB balances are associated with higher cash holdings in my samples (untabulated).

35 I classify firms with a year‐end UTB balance in the top annual quartile as reporting high UTB reserves (*HighUTB*). Consistent with Hanlon et al. (2017), I scale UTB balances by total assets. Moreover, I limit the sample to firms with positive year‐end UTB balances to address concerns about misreported UTB values.

36 I use a Chow (1960) test to test for differences in the coefficients on *CVTax* in Table 6 and Table S3 in supporting information in the online Appendix.

37 Because dividend payouts in year *t* affect retained earnings in the same year, I require a firm to fulfill this requirement in two consecutive years.

38 For brevity, I only report marginal effects for the logit regressions in Tables 7 and 9.

39 In untabulated tests, I do not orthogonalize *CVTax* but accommodate loss observations following Guenther et al. (2017). Specifically, I require a positive numerator and a positive denominator when calculating annual cash ETRs. The results are consistent with my main findings.

40 I follow the approach for the primary analysis and again orthogonalize the alternative measures of the volatility of tax payments used in Table 7.

41 In contrast to the coefficient of variation, the standard deviation is sensitive to the scale of the underlying variable. This can impede the comparison of effect sizes of variables with different scales (Sørensen 2002). I therefore use *SDTax* in a robustness test and prefer the coefficient of variation for annual cash ETRs (*CVTax*) as a primary measure.

42 I use nonorthogonalized values of *CVTax* because these tests provide alternative approaches to controlling for operating risk.

43 Entropy balancing reweighs observations to adjust for differences in firm characteristics along the first (mean), the second (variance), and the third (skewness) moment of the variable distribution (Hainmueller 2012). A limitation of this method is that limited overlap between dividend‐paying and non‐dividend‐paying firms could produce extreme balancing weights. Results are similar when dropping observations with balancing weights in the 99th percentile (untabulated).

44 I conduct one‐to‐one nearest neighbor matching without replacement and applying a caliper of 1.5%. To match observations per year and Fama and French 17 industry codes, I estimate propensity scores for each industry‐year with at least 90 observations. Results in Table S5 in supporting information in the online Appendix suggest that the matching achieves covariate balance between dividend‐paying and non‐dividend‐paying firms. Moreover, standardized differences of covariates (untabulated) are well below the cutoff value of 0.25 (Stuart et al. 2013). I show in supporting information in the online Appendix (Table S6) that the results are robust to alternative matching approaches.

45 This approach estimates the effect of Δ *CVTax* on dividend omissions (*DivOmit*) and dividend initiations (*DivInitiate*) relative to a base group of firms that do not change their dividend payouts. These firms either distribute dividends or do not distribute dividends in the years *t* − 1 and *t*. In untabulated tests, I estimate two separate logit regressions to examine the effect of Δ *CVTax* on (i) dividend omissions relative to a base group of firms that distribute dividends in the years *t* − 1 and *t* and (ii) dividend initiations relative to a base group of firms that do not distribute dividends in either year. The results are consistent with the findings in supporting information in the online Appendix (Table S7).

46 I do not include firm fixed effects in this test because a changes specification represents an alternative (yet very similar) approach to controlling for omitted time‐invariant firm characteristics (Wooldridge 2010, 314).

47 The tests in Table 8 are designed to exploit between‐firm differences in the relation between volatile tax payments and variation in future after‐tax cash flows. I therefore include industry instead of firm fixed effects.

48 The volatility of operating cash flows (sales) is associated with more (less) volatile future after‐tax cash flows. The positive coefficient on *CVSales* suggests that variation in sales might capture a growth component, being associated with less volatile future after‐tax cash flows as firms mature (DeAngelo et al. 2006).

49 I include industry instead of firm fixed effects in Table 9 (panel B) and Table S8 in supporting information in the online Appendix, because these tests are designed to examine *between*‐firm differences in the association between the volatility of tax payments and dividend payouts.

50 To allow a comparison with the descriptive statistics in Table 2, I present nonorthogonalized values of *CVTax* in supporting information in the online Appendix (Tables S9 and S10). Inferences are unchanged when using orthogonalized values of *CVTax* (untabulated).

51 I follow Hanlon and Hoopes (2014) and obtain dividend distribution data from CRSP. I classify distribution codes between 1260 and 1299 as special dividends.

## Variable definitions

**Table:**

Variable	Description
**Dependent variables**	
*DivPay*	Indicator variable equal to one if a firm declares dividends on common stock in year *t* (DVC_*t*_ > 0), and zero otherwise (DVC_*t*_ = 0)
*DivAmount*	Natural logarithm of dividends declared on common stock in year *t* (DVC_*t*_) divided by total assets in year *t* (AT_*t*_)
*DivOmit*	Indicator variable equal to one if a firm does not declare dividends in year *t* (DVC_*t*_ = 0) but did declare dividends in year *t* − 1 (DVC_*t*−1_ > 0). The variable is zero if a firm does not change its dividend payouts from year *t* − 1 to year *t*
*DivInitiate*	Indicator variable equal to one if a firm declares dividends in year *t* (DVC_*t*_ > 0) but did not declare dividends in year *t* − 1 (DVC_*t*−1_ = 0). The variable is zero if a firm does not change its dividend payouts from year *t* − 1 to year *t*
*CVTotalCF*	Coefficient of variation for the ratio of net operating cash flows in year *t* (OANCF_t_) divided by total assets in year *t* − 1 (AT_*t*−1_) over the five‐year period *t* + 1 to *t* + 5
*RepuPay*	Indicator variable equal to one if a firm repurchases shares in year *t*, and zero otherwise. I follow Grullon and Michaely (2002) and compute share repurchases as purchases of common and preferred stock in year *t* (PRSTKC_*t*_) less the change in the redemption value of preferred stock in year *t* (PSTKRV_*t*−1_ – PSTRKV_*t*_)
*SpecialDivPay*	Indicator variable equal to one if a firm distributes special dividends in year *t*, and zero otherwise. I follow Hanlon and Hoopes (2014) and classify distribution codes between 1260 and 1299 as special dividends (DISTCD_*t*_). I obtain monthly special‐dividend data from CRSP
**Measures of the volatility of tax payments**	
*CVTax*	Coefficient of variation for annual cash ETRs over the five‐year period *t* − 4 to *t*. I calculate annual cash ETRs in year *t* as cash taxes paid in year *t* (TXPD_*t*_) divided by pre‐tax income in year *t* (PI_*t*_) adjusted for special items (SPI_*t*_). I set missing values for SPI_*t*_ to zero. I require nonmissing values in each year. I set annual cash ETRs to 1 in case of a positive numerator and a negative denominator and to 0 in case of a negative numerator and a negative denominator. I winsorize annual cash ETRs at 1 and 0
*RCVTax*	Decile rank of *CVTax* in year *t* within deciles of *CVCashFlow* in year *t* (*RCVCashFlow*)
*SDTax*	Standard deviation of annual cash ETRs over the five‐year period *t* − 4 to *t*. I calculate annual cash ETRs in year *t* as cash taxes paid in year *t* (TXPD_t_) divided by pre‐tax income in year *t* (PI_*t*_) adjusted for special items (SPI_*t*_). I set missing values for SPI_*t*_ to zero. I require nonmissing values in each year. I set annual cash ETRs to 1 in case of a positive numerator and a negative denominator and to 0 in case of a negative numerator and a negative denominator. I winsorize annual cash ETRs at 1 and 0
*HighTaxVol*	Indicator variable equal to one if the annual cash ETR and the average annual cash ETR are larger than 0.6 in any of the years *t* − 4 to *t*, and zero otherwise. I follow Saavedra (2019) and calculate average annual cash ETRs as cash taxes paid in year *t* (TXPD_*t*_) divided by the average pre‐tax income over the three‐year period *t* − 2 to *t* (PI_*t*−2;*t*_) adjusted for special items (SPI_*t*−2;*t*_). I require a positive denominator and nonmissing values in each year
*GAAPCVTax*	Coefficient of variation for annual GAAP ETRs over the five‐year period *t* − 4 to *t*. I calculate annual GAAP ETRs in year *t* as tax expense in year *t* (TXT_*t*_) divided by pre‐tax income in year *t* (PI_*t*_) adjusted for special items (SPI_*t*_). I set missing values for SPI_*t*_ to zero. I require nonmissing values in each year. I set annual GAAP ETRs to 1 in case of a positive numerator and a negative denominator and to 0 in case of a negative numerator and a negative denominator. I winsorize annual GAAP ETRs at 1 and 0
**Control variables**	
*CashETR*	*CashETR* is the sum of cash taxes paid over the five‐year period *t* − 4 to *t* (TXPD_*t*−4;*t*_) divided by the sum of pre‐tax income over the five‐year period *t* − 4 to *t* (PI_*t*−4;*t*_) adjusted for special items (SPI_*t*−4;*t*_). I set missing values for SPI_*t*_ to zero. I follow Dyreng et al. (2008) and require a positive denominator. I winsorize *CashETR* at 1 and 0
*TaxAvoidance*	Tax avoidance calculated by multiplying *CashETR* by negative one in all regressions
*CashFlow*	Net operating cash flow in year *t* (OANCF_*t*_) adjusted for cash taxes paid in year *t* (TXPD_*t*_) divided by total assets in year *t* − 1 (AT_*t*−1_)
*CVCashFlow*	Coefficient of variation for *CashFlow* over the five‐year period *t* − 4 to *t*
*CVRoa*	Coefficient of variation for the ratio of pre‐tax income in year *t* (PI_*t*_) divided by total assets in year *t* − 1 (AT_*t*−1_) over the five‐year period *t* − 4 to *t*
*CVSales*	Coefficient of variation for the ratio of sales in year *t* (SALE_*t*_) divided by total assets in year *t* − 1 (AT_*t*−1_) over the five‐year period *t* − 4 to *t*
*Cash*	Cash and short‐term investments in year *t* (CHE_*t*_) divided by total assets in year *t* (AT_*t*_)
*MTB*	Market value of equity in year *t* (PRCC_F_*t*_ × CSHO_*t*_) divided by book value of equity in year *t* (CEQ_*t*_)
*SalesGrowth*	Sales in year *t* (SALE_*t*_) divided by sales in year *t* − 2 (SALE_*t*−2_) less 1
*AssetGrowth*	Total assets in year *t* (AT_*t*_) divided by total assets in year *t* − 1 (AT_*t*−1_) less 1
*RETE*	Retained earnings in year *t* (RE_*t*_) divided by shareholder equity in year *t* (SEQ_*t*_)
*Age*	Natural logarithm of 1 plus firm age in year *t*. I follow Hadlock and Pierce (2010) and compute firm age as year *t* (FYEAR_*t*_) less the first firm‐year with a non‐missing stock price (PRCC_F_*t*_) in Compustat. I set negative values for firm age to zero
*Size*	Natural logarithm of total assets in year *t* (AT_*t*_)
*Leverage*	Long‐term debt in year *t* (DLTT_t_) divided by total assets in year *t* (AT_*t*_)
*PCM*	Price–cost margin in year *t*. I follow Kubick et al. (2015) and compute *PCM* as operating profit in year *t* (SALE_*t*_ – COGS_*t*_ – XSGA_*t*_) divided by sales in year *t* (SALE_*t*_) less the sales‐weighted industry average price–cost margin in year *t* based on the full sample and Fama and French 17 industry codes. I use operating income after depreciation in year *t* (OIADP_*t*_) as a measure of operating profit if COGS_*t*_ or XSGA_*t*_ are missing
*Loss*	Indicator variable equal to one if a firm reports negative net income in year *t* (NI_*t*_ < 0), and zero otherwise
*NOL*	Amount of net operating loss carryforward in year *t* (TLCF_*t*_) divided by total assets in year *t* − 1 (AT_*t*−1_). I set missing values to zero
*R&D*	Research and development expense in year *t* (XRD_*t*_) divided by total assets in year *t* − 1 (AT_*t*−1_). I set missing values to zero
*SGA*	Selling, general, and administrative expense in year *t* (XSGA_*t*_) divided by total assets in year *t* − 1 (AT_*t*−1_). I set missing values to zero
*Advertising*	Advertising expense in year *t* (XAD_*t*_) divided by total assets in year *t* − 1 (AT_*t*−1_). I set missing values to zero
*CapIntensity*	Property, plant, and equipment in year *t* (PPEGT_*t*_) divided by total assets in year *t* − 1 (AT_*t*−1_)
**Additional variables**	
*LossFirm*	Indicator variable equal to one if a firm reports negative pre‐tax income (PI_*t*_) adjusted for special items (SPI_*t*_) in at least one of the years between *t* − 4 to *t*, and zero otherwise
*FactorScore*	Factor score obtained from a principal component analysis of *CVCashFlow* in year *t*, *CVRoa* in year *t*, and *CVSales* in year *t*
*RCVCashFlow*	Decile rank of *CVCashFlow* in year *t*
*SDCashFlow*	Standard deviation of *CashFlow* over the five‐year period *t* − 4 to *t*
*SDRoa*	Standard deviation of the ratio of pre‐tax income in year *t* (PI_*t*_) divided by total assets in year *t* − 1 (AT_*t*−1_) over the five‐year period *t* − 4 to *t*
*SDSales*	Standard deviation of the ratio of sales in year *t* (SALE_*t*_) divided by total assets in year *t* − 1 (AT_*t*−1_) over the five‐year period *t* − 4 to *t*
*GAAPETR*	Sum of tax expense over the five‐year period *t* − 4 to *t* (TXT_*t*−4;*t*_) divided by the sum of pre‐tax income over the five‐year period *t* − 4 to *t* (PI_*t*−4;*t*_) adjusted for special items (SPI_*t*−4;*t*_). I set missing values for SPI_*t*_ to zero. I follow Dyreng et al. (2008) and require a positive denominator. I winsorize *GAAPETR* at 1 and 0
*TaxAvoidanceGAAP*	Tax avoidance calculated by multiplying *GAAPETR* by negative one in all regressions
**Partitioning variables**	
*HighInst*	Indicator variable equal to one if the share of mean institutional ownership in year *t* (INSTOWN_PERC_*t*_) is in the top annual quartile, and zero otherwise. I obtain ownership data from the Thomson Reuters Institutional (13f) Holdings Database
*HighUTB*	Indicator variable equal to one if the ratio of the ending balance of the UTB reserve (TXTUBEND_t_) divided by total assets in year *t* is in the top annual quartile, and zero otherwise
*Rating*	Indicator variable equal to one if the firm does not have an S&P Issuer Credit Rating in year *t* (SPLTICRM_*t*_), and zero otherwise
*LowRETE*	Indicator variable equal to one if the ratio of retained earnings divided by shareholder equity in year *t* (*RETE*) is in the bottom annual quartile for two consecutive years, and zero otherwise

*Notes*: I provide Compustat variable names in parentheses. Variables collected from other sources are defined accordingly.
