# Supplementary material for: Volatility of Tax Payments and Dividend Payouts
Source: Contemp Account Res. 2022 Dec 31;40(1):451–87. doi: 10.1111/1911-3846.12831 (PMC10962253; doi:10.1111/1911-3846.12831)
Supplement: Supplementary file 1 — Online Appendix. Supporting information [file CARE-40-451-s001.docx]

**Online Appendix for**

**“Volatility of Tax Payments and Dividend Payouts”**

HARALD J. AMBERGER, *Vienna University of Economics and Business*

| TABLE S1 | | | | | | |
| --- | --- | --- | --- | --- | --- | --- |
| Summary statistics for the orthogonalization of *CVTax* | | | | | | |
| **Panel A:** Full sample |  |  |  |  |  |  |
|  | (1) | (2) | (3) | (4) | (5) | (6) |
|  | Mean | SD | 25% | Median | 75% | Percent > 0 |
| Coefficients on *CVCashFlow* | 0.039 | 0.124 | -0.007 | 0.036 | 0.082 | 71.76% |
| Coefficients on *CVRoa* | 0.012 | 0.047 | -0.005 | 0.004 | 0.017 | 62.75% |
| Coefficients on *CVSales* | 0.385 | 0.489 | 0.127 | 0.397 | 0.622 | 84.31% |
| Coefficients on *LossFirm* | 0.509 | 0.185 | 0.416 | 0.511 | 0.623 | 98.43% |
| *Intercept* | 0.436 | 0.139 | 0.345 | 0.407 | 0.502 | 100.00% |
| Adjusted R² | 0.402 | 0.133 | 0.309 | 0.391 | 0.487 |  |
|  |  |  |  |  |  |  |
| **Panel B:** Subsample of dividend-paying firms | | | |  |  |  |
|  | (1) | (2) | (3) | (4) | (5) | (6) |
|  | Mean | SD | 25% | Median | 75% | Percent > 0 |
| Coefficients on *CVCashFlow* | 0.183 | 0.324 | -0.011 | 0.165 | 0.288 | 74.12% |
| Coefficients on *CVRoa* | 0.077 | 0.160 | -0.005 | 0.028 | 0.096 | 70.98% |
| Coefficients on *CVSales* | 0.423 | 0.940 | -0.068 | 0.335 | 0.920 | 72.16% |
| Coefficients on *LossFirm* | 0.367 | 0.441 | 0.239 | 0.399 | 0.591 | 90.20% |
| *Intercept* | 0.291 | 0.166 | 0.182 | 0.273 | 0.373 | 96.86% |
| Adjusted R² | 0.469 | 0.178 | 0.334 | 0.456 | 0.596 |  |
| *Notes:* This table presents summary statistics for the regression coefficients on the independent variables used in orthogonalizing *CVTax*. Panel A (B) presents summary statistics for the full sample (the subsample). I orthogonalize *CVTax* with respect to *CVCashFlow*, *CVRoa*, *CVSales*, and *LossFirm* per year and Fama and French 17 industry codes. The full sample and the subsample each enter the orthogonalization procedure with 255 unique industry-year combinations. Variables are defined in the Appendix. | | | | | | |

| TABLE S2 | | | | | | |
| --- | --- | --- | --- | --- | --- | --- |
| Cross-sectional evidence: Volatility of tax payments and borrowing costs | | | | | | |
|  |  | (1) | (2) |  | (3) | (4) |
|  |  | Coef. (SE) | ME |  | Coef. (SE) | ME |
| Subsample |  | *Rating* = 1 | |  | *Rating* = 0 | |
|  |  | *Low Borrowing Costs* | |  | *High Borrowing Costs* | |
| Dependent variable |  | *DivPay* | |  | *DivPay* | |
| *CVTax* |  | -0.129*** | -0.030 |  | -0.254*** | -0.048 |
|  |  | (0.048) |  |  | (0.038) |  |
| Controls |  | Y | |  | Y | |
| Year FE |  | Y | |  | Y | |
| Industry FE |  | Y | |  | Y | |
| Observations |  | 10,915 | |  | 22,108 | |
| Pseudo R² |  | 0.305 | |  | 0.243 | |
| *CVTax* (1) > (3)  p-value (one-tailed) |  | 0.035 | | | | |
| *Notes:* This table presents regression results for a cross-sectional test based on a firm’s borrowing costs for the full sample. I classify a firm without a Standard & Poor's Issuer Credit Rating in year *t* as having high borrowing costs. Columns (1) and (3) (columns (2) and (4)) report coefficients (marginal effects) for a logit regression based on equation (1). I calculate marginal effects while holding continuous variables at their means. All variables are standardized to have a mean of zero and a standard deviation of one in each subsample prior to fitting regressions. All regressions are estimated with year and industry fixed effects. I report heteroscedasticity-robust standard errors clustered by firm in parentheses. Variables are defined in the Appendix. *** represent significance levels of 0.01 (two-tailed). | | | | | | |

| TABLE S3 | | | | | |
| --- | --- | --- | --- | --- | --- |
| Cross-sectional evidence: Volatility of tax payments and costs of dividend reductions | | | | | |
|  |  | (1) |  | (2) | |
|  |  | Coef. (SE) |  | Coef. (SE) | |
| Subsamples |  | *LowRETE* = 1 |  | *LowRETE* = 0 | |
|  |  | *Growing* |  | *Mature* | |
| Dependent variable |  | *DivAmount* |  | *DivAmount* | |
| *CVTax* |  | 0.013 |  | -0.022** | |
|  |  | (0.018) |  | (0.011) | |
| Controls |  | Y |  | Y | |
| Year FE |  | Y |  | Y | |
| Firm FE |  | Y |  | Y | |
| Observations |  | 2,455 |  | 8,748 | |
| Adjusted R² |  | 0.206 |  | 0.161 | |
| *CVTax* (1) > (2)  p-value (one-tailed) |  | 0.020 | | | |
| *Notes:* This table presents regression results for a cross-sectional test based on the costs of dividend reductions for the subsample. I identify a firm with a ratio of retained earnings divided by shareholder equity in the bottom annual quartile for two consecutive years as incurring low costs for dividend reductions. All columns report coefficients for a linear regression based on equation (1). All variables are standardized to have a mean of zero and a standard deviation of one in each subsample prior to fitting regressions. All regressions are estimated with year and firm fixed effects. I report heteroscedasticity-robust standard errors clustered by industry in parentheses. Variables are defined in the Appendix. ** represent significance levels of 0.05 (two-tailed). | | | | |  |

| TABLE S4 | | | | |  |  |  |  |  |  |
| --- | --- | --- | --- | --- | --- | --- | --- | --- | --- | --- |
| Controlling for operating risk and differences in firm characteristics | | | | | | | | | |  |
|  |  | (1) |  | (2) |  | (3) |  |  | (4) |  |
|  |  | ME (SE) |  | ME (SE) |  | ME (SE) |  |  | Coef. (SE) |  |
| Analysis |  | Decile Ranks |  | Entropy Balancing |  | PSM |  |  | Decile Ranks |  |
| Dependent variable |  | *DivPay* |  | *DivPay* |  | *DivPay* |  |  | *DivAmount* |  |
| *RCVTax* |  | -0.070*** |  |  |  |  |  |  | -0.049*** |  |
|  |  | (0.007) |  |  |  |  |  |  | (0.010) |  |
| *RCVCashFlow* |  | -0.068*** |  |  |  |  |  |  | -0.079*** |  |
|  |  | (0.008) |  |  |  |  |  |  | (0.012) |  |
| *CVTax* |  |  |  | -0.039*** |  | -0.076*** |  |  |  |  |
|  |  |  |  | (0.014) |  | (0.011) |  |  |  |  |
| *CVCashFlow* |  |  |  | -0.003 |  | 0.004 |  |  |  |  |
|  |  |  |  | (0.011) |  | (0.007) |  |  |  |  |
| *CVRoa* |  | 0.000 |  | 0.001 |  | 0.006 |  |  | -0.001 |  |
|  |  | (0.004) |  | (0.008) |  | (0.006) |  |  | (0.010) |  |
| *CVSales* |  | -0.035*** |  | -0.003 |  | 0.011 |  |  | -0.015 |  |
|  |  | (0.008) |  | (0.012) |  | (0.009) |  |  | (0.012) |  |
| Additional Controls |  | Y |  | Y |  | Y |  |  | Y |  |
| Year FE |  | Y |  | Y |  | Y |  |  | Y |  |
| Industry FE |  | Y |  | Y |  | Y |  |  | N |  |
| Firm FE |  | N |  | N |  | N |  |  | Y |  |
| Observations |  | 33,023 |  | 33,023 |  | 7,868 |  |  | 13,694 |  |
| Pseudo/Adjusted R² |  | 0.298 |  | 0.042 |  | 0.012 |  |  | 0.172 |  |
| *Notes:* This table presents regression results for tests that control for operating risk and differences in firm characteristics. Columns (1-3) present results based on the full sample. Column (4) presents results based on the subsample. Columns (1-3) report marginal effects for a logit regression based on equation (1). Column (4) reports coefficients for a linear regression based on equation (1). In columns (1) and (4), I sort observations into deciles of *CVTax* within deciles of *CVCashFlow*. In column (2) (column (3)), I employ entropy balancing (propensity score matching) to match dividend-paying with non-dividend-paying firms. I conduct one-to-one nearest neighbor matching without replacement, applying a caliper of 1.5 percent. I require exact matches per year and Fama and French 17 industry codes. I calculate marginal effects while holding continuous variables at their means. All variables are standardized to have a mean of zero and a standard deviation of one prior to fitting regressions. In columns (1-3), regressions are estimated with year and industry fixed effects. The regression in column (4) is estimated with year and firm fixed effects. When estimating the regression in column (2), I weigh observations by the frequency weights obtained from entropy balancing. In columns (1-3) (column (4)), I report heteroscedasticity-robust standard errors clustered by firm (industry) in parentheses. Variables are defined in the Appendix. *** represent significance levels of 0.01 (two-tailed). | | | | | | | | | | |

| TABLE S5 | | | |  |  |
| --- | --- | --- | --- | --- | --- |
| Covariate balance after propensity score matching | | | |  |  |
|  | *DivPay* = 1 |  | *DivPay* = 0 |  | Differences |
|  | Firms-Years  (N = 3,934) |  | Firms-Years  (N = 3,934) |  | p-Values |
|  | Mean |  | Mean |  | *t*-Test |
| *TaxAvoidance* | 0.283 |  | 0.284 |  | 0.896 |
| *CashFlow* | 0.150 |  | 0.151 |  | 0.841 |
| *CVCashFlow* | 0.463 |  | 0.470 |  | 0.704 |
| *CVRoa* | 0.639 |  | 0.604 |  | 0.681 |
| *CVSales* | 0.169 |  | 0.170 |  | 0.812 |
| *Cash* | 0.155 |  | 0.156 |  | 0.895 |
| *MTB* | 2.679 |  | 2.698 |  | 0.733 |
| *SalesGrowth* | 0.201 |  | 0.212 |  | 0.183 |
| *AssetGrowth* | 0.097 |  | 0.100 |  | 0.585 |
| *RETE* | 0.490 |  | 0.476 |  | 0.448 |
| *Age* | 2.887 |  | 2.907 |  | 0.066* |
| *Size* | 6.348 |  | 6.394 |  | 0.232 |
| *Leverage* | 0.162 |  | 0.159 |  | 0.483 |
| *PCM* | -0.018 |  | -0.018 |  | 0.922 |
| *Loss* | 0.122 |  | 0.126 |  | 0.599 |
| *NOL* | 0.046 |  | 0.051 |  | 0.224 |
| *R&D* | 0.025 |  | 0.026 |  | 0.441 |
| *SGA* | 0.283 |  | 0.283 |  | 0.892 |
| *Advertising* | 0.012 |  | 0.011 |  | 0.728 |
| *CapIntensity* | 0.520 |  | 0.529 |  | 0.250 |
| *Notes:* This table assesses the covariate balance for the propensity score matched sample in column (3) of Table 5. I conduct one-to-one nearest neighbor matching of dividend-paying firms (*DivPay* = 1) and non-dividend-paying firms (*DivPay* = 0) without replacement and apply a caliper of 1.5 percent. I require exact matches per year and Fama and French 17 industry codes. I conduct matched pairs t-tests to compare means between subsamples. Variables are defined in the Appendix. * represents significance levels of 0.10 (two-tailed). | | | | | |

| TABLE S6 | | | | |  |  |
| --- | --- | --- | --- | --- | --- | --- |
| Alternative matching approaches | | | | |  |  |
|  |  | (1) |  | (2) |  | (3) |
|  |  | ME (SE) |  | ME (SE) |  | ME (SE) |
| Analysis |  | PSM Replacement |  | PSM Caliper  5 Percent |  | Simple Matching |
| Dependent variable |  | *DivPay* |  | *DivPay* |  | *DivPay* |
| *CVTax* |  | -0.078*** |  | -0.080*** |  | -0.063*** |
|  |  | (0.013) |  | (0.011) |  | (0.011) |
| Controls |  | Y |  | Y |  | Y |
| Year FE |  | Y |  | Y |  | Y |
| Industry FE |  | Y |  | Y |  | Y |
| Observations |  | 11,652 |  | 8,384 |  | 9,572 |
| Pseudo R² |  | 0.014 |  | 0.014 |  | 0.189 |
| *Notes:* This table presents regression results for alternative approaches to match dividend-paying with non-dividend-paying firms based on the full sample. All columns report marginal effects for a logit regression based on equation (1). In column (1), I conduct one-to-one nearest neighbor propensity score matching with replacement, applying a caliper of 1.5 percent. In column (2), I match without replacement, applying a caliper of 5 percent. In column (3), I match observations without replacement on *CVCashFlow* using Mahalanobis distance matching. I require *CVCashFlow* to match within a band of +/- 1.5 percent. I calculate marginal effects while holding continuous variables at their means. All variables are standardized to have a mean of zero and a standard deviation of one prior to fitting regressions. All regressions are estimated with year and industry fixed effects. When estimating the regressions in column (1), I weight observations by their frequency in the sample. I report heteroscedasticity-robust standard errors clustered by firm in parentheses. Variables are defined in the Appendix. *** represent significance levels of 0.01 (two-tailed). | | | | | | |

| TABLE S7 | | | | | | | |
| --- | --- | --- | --- | --- | --- | --- | --- |
| Changes specification | | | | | | | |
|  |  | (1) |  | (2) |  |  | (3) |
|  |  | ME (SE) |  | ME (SE) |  |  | Coef. (SE) |
| Analysis |  | Changes |  | Changes |  |  | Changes |
| Dependent variable |  | *DivOmit* |  | *DivInitiate* |  |  | Δ *DivAmount* |
| Δ *CVTax* |  | 0.003*** |  | -0.001 |  |  | -0.010** |
|  |  | (0.001) |  | (0.001) |  |  | (0.005) |
| Δ Controls |  | Y |  | Y |  |  | Y |
| Year FE |  | Y |  | Y |  |  | Y |
| Industry FE |  | Y |  | Y |  |  | Y |
| Observations |  | 27,772 | | |  |  | 11,410 |
| Pseudo/Adjusted R² |  | 0.054 | | |  |  | 0.132 |
| *Notes:* This table presents regression results for estimating equation (1) as a changes specification. Columns (1-2) present results for the full sample. Column (3) presents results for the subsample. Columns (1-2) report marginal effects for a multinomial logistic regression. Column (3) reports coefficients for a linear regression. I calculate marginal effects while holding continuous variables at their means. Changes for variables are measured from year *t-1* to *t*. All variables are standardized to have a mean of zero and a standard deviation of one prior to fitting regressions. All regressions are estimated with year and industry fixed effects. I report heteroscedasticity-robust standard errors clustered by firm in parentheses. Variables are defined in the Appendix. ** and *** represent significance levels of 0.05 and 0.01, respectively (two-tailed). | | | | | | | |

| TABLE S8 | | | | | | |  |  |  |  |
| --- | --- | --- | --- | --- | --- | --- | --- | --- | --- | --- |
| Volatility of tax payments and future dividend payouts | | | | | | | |  |  |  |
| **Panel A:** The probability of dividend payouts | | | | | | | | |  |  |
|  |  | (1) |  | (2) |  | (3) |  | (4) |  | (5) |
|  |  | ME (SE) |  | ME (SE) |  | ME (SE) |  | ME (SE) |  | ME (SE) |
| Dependent variable |  | *DivPay_t+1* |  | *DivPay_t+2* |  | *DivPay_t+3* |  | *DivPay_t+4* |  | *DivPay_t+5* |
| *CVTax* |  | -0.028*** |  | -0.038*** |  | -0.039*** |  | -0.034*** |  | -0.037*** |
|  |  | (0.007) |  | (0.008) |  | (0.008) |  | (0.008) |  | (0.009) |
| *DivPay_t* |  | 1.529*** |  | 1.320*** |  | 1.157*** |  | 1.024*** |  | 0.907*** |
|  |  | (0.023) |  | (0.022) |  | (0.021) |  | (0.022) |  | (0.022) |
| Year FE |  | Y |  | Y |  | Y |  | Y |  | Y |
| Industry FE |  | Y |  | Y |  | Y |  | Y |  | Y |
| Observations |  | 27,772 |  | 23,727 |  | 20,426 |  | 17,628 |  | 15,186 |
| Pseudo R² |  | 0.742 |  | 0.632 |  | 0.549 |  | 0.484 |  | 0.431 |

| **Panel B:** The amount of dividend payouts | | | | | | | | |  |  |
| --- | --- | --- | --- | --- | --- | --- | --- | --- | --- | --- |
|  |  | (1) |  | (2) |  | (3) |  | (4) |  | (5) |
|  |  | Coef. (SE) |  | Coef. (SE) |  | Coef. (SE) |  | Coef. (SE) |  | Coef. (SE) |
| Dependent variable |  | *DivAmount_t+1* |  | *DivAmount_t+2* |  | *DivAmount_t+3* |  | *DivAmount_t+4* |  | *DivAmount_t+5* |
| *CVTax* |  | -0.018*** |  | -0.020*** |  | -0.029*** |  | -0.036*** |  | -0.042*** |
|  |  | (0.005) |  | (0.007) |  | (0.009) |  | (0.012) |  | (0.014) |
| *DivAmount_t* |  | 0.859*** |  | 0.816*** |  | 0.774*** |  | 0.725*** |  | 0.689*** |
|  |  | (0.010) |  | (0.012) |  | (0.014) |  | (0.018) |  | (0.022) |
| Year FE |  | Y |  | Y |  | Y |  | Y |  | Y |
| Industry FE |  | Y |  | Y |  | Y |  | Y |  | Y |
| Observations |  | 10,777 |  | 9,238 |  | 7,984 |  | 6,938 |  | 6,027 |
| Adjusted R² |  | 0.776 |  | 0.713 |  | 0.651 |  | 0.586 |  | 0.533 |
| *Notes:* This table presents regression results for tests that examine the relation between the volatility of tax payments and future dividend payouts. Panel A (B) includes tests based on the full sample (the subsample). Panel A (B) reports marginal effects for a logit regression (coefficients for a linear regression) of future dividend payouts regressed on dividend payouts in year *t* and the volatility of tax payments in year *t* (*CVTax*). I calculate marginal effects while holding continuous variables at their means. All variables are standardized to have a mean of zero and a standard deviation of one in each subsample prior to fitting regressions. All regressions are estimated with year and industry fixed effects. I report heteroscedasticity-robust standard errors clustered by firm in parentheses. Variables are defined in the Appendix. *** represent significance levels of 0.01 (two-tailed). | | | | | | | | | | |

| TABLE S9 | | | | | | |
| --- | --- | --- | --- | --- | --- | --- |
| Volatility of tax payments and share repurchases | | | | |  |  |
| **Panel A:** *DivPay* =0, *RepuPay* = 0 | | | | | | |
|  | (1) | (2) | (3) | (4) | (5) | (6) |
|  | N | Mean | SD | 25% | Median | 75% |
| *CVTax* | 9,390 | 0.904 | 0.511 | 0.523 | 0.839 | 1.211 |
|  |  |  |  |  |  |  |
| **Panel B:** *DivPay* =0, *RepuPay* = 1 | | | | | | |
|  | (1) | (2) | (3) | (4) | (5) | (6) |
|  | N | Mean | SD | 25% | Median | 75% |
| *CVTax* | 8,727 | 0.757 | 0.482 | 0.378 | 0.673 | 1.045 |
|  |  |  |  |  |  |  |
| **Panel C:** *DivPay* =1, *RepuPay* = 0 | | |  |  |  |  |
|  | (1) | (2) | (3) | (4) | (5) | (6) |
|  | N | Mean | SD | 25% | Median | 75% |
| *CVTax* | 4,549 | 0.648 | 0.457 | 0.286 | 0.570 | 0.901 |
|  |  |  |  |  |  |  |
| **Panel D:** *DivPay* =1, *RepuPay* = 1 | | |  |  |  |  |
|  | (1) | (2) | (3) | (4) | (5) | (6) |
|  | N | Mean | SD | 25% | Median | 75% |
| *CVTax* | 8,751 | 0.482 | 0.381 | 0.198 | 0.366 | 0.669 |
| *Notes:* This table presents descriptive statistics for the volatility of tax payments conditional on a firm’s dividend payouts and share repurchases. Panel A presents information for firms that neither distribute dividends nor repurchase shares in year *t*, panel B (C) for firms that repurchase shares (distribute dividends) in year *t*, and panel D for firms that repurchase shares and distribute dividends in year *t*. Variables are defined in the Appendix. | | | | | | |

| TABLE S10 | | | | | | |
| --- | --- | --- | --- | --- | --- | --- |
| Volatility of tax payments and special dividends | | | | |  |  |
| **Panel A:** *DivPay* =0, *SpecialDivPay* = 0 | | | | | | |
|  | (1) | (2) | (3) | (4) | (5) | (6) |
|  | N | Mean | SD | 25% | Median | 75% |
| *CVTax* | 19,249 | 0.836 | 0.504 | 0.448 | 0.765 | 1.139 |
|  |  |  |  |  |  |  |
| **Panel B:** *DivPay* =0, *SpecialDivPay* = 1 | | | | | | |
|  | (1) | (2) | (3) | (4) | (5) | (6) |
|  | N | Mean | SD | 25% | Median | 75% |
| *CVTax* | 252 | 0.672 | 0.447 | 0.299 | 0.588 | 0.950 |
|  |  |  |  |  |  |  |
| **Panel C:** *DivPay* =1, *SpecialDivPay* = 0 | | |  |  |  |  |
|  | (1) | (2) | (3) | (4) | (5) | (6) |
|  | N | Mean | SD | 25% | Median | 75% |
| *CVTax* | 12,000 | 0.529 | 0.411 | 0.216 | 0.409 | 0.735 |
|  |  |  |  |  |  |  |
| **Panel D:** *DivPay* =1, *SpecialDivPay* = 1 | | |  |  |  |  |
|  | (1) | (2) | (3) | (4) | (5) | (6) |
|  | N | Mean | SD | 25% | Median | 75% |
| *CVTax* | 311 | 0.523 | 0.434 | 0.198 | 0.371 | 0.722 |
| *Notes:* This table presents descriptive statistics for the volatility of tax payments conditional on a firm’s regular and special dividend payouts. Panel A presents information for firms that neither distribute regular nor special dividends in year *t*, panel B (C) for firms that distribute special dividends (regular dividends) in year *t*, and panel D for firms that distribute regular and special dividends in year *t*. Variables are defined in the Appendix. | | | | | | |
